# Diagnostic Accuracy of Cardiovascular Disease Prediction Models: A Systematic Review and Meta‐Analysis of Validation Studies

**DOI:** 10.1002/hsr2.72469

**Published:** 2026-04-29

**Authors:** Mohammad Aziz Rasouli, Farid Najafi, Alireza Ansari‐Moghaddam, Bijan Nouri, Farhad Moradpour, Ghobad Moradi, Yousef Moradi

**Affiliations:** ^1^ Social Determinant of the Health Research Center, Research, Institute for Health Development Kurdistan University of Medical Sciences Sanandaj Iran; ^2^ Department of Epidemiology and Biostatistics, School of Medicine Kurdistan University of Medical Sciences Sanandaj Iran; ^3^ Research Center for Environmental Determinants of Health, Health Institute Kermanshah University of Medical Sciences Kermanshah Iran; ^4^ Health Promotion Research Center Zahedan University of Medical Sciences Zahedan Iran; ^5^ Health Metrics and Evaluation Research Center, Research Institute for Health Development Kurdistan University of Medical Sciences Sanandaj Iran; ^6^ Center for Communicable Disease Control Iran Ministry of Health and Medical Education Tehran Iran

**Keywords:** accuracy, cardiovascular diseases, evidence synthesis, prediction model

## Abstract

**Background and Aim:**

Cardiovascular diseases (CVD) are widely accepted to be the most serious health care problem in the world. We performed a diagnostic test accuracy systematic review and meta‐analysis to establish the real‐world performance of established CVD risk prediction tools, providing high‐certainty evidence to optimize primary prevention strategies in resource‐constrained healthcare systems worldwide.

**Methods:**

Databases including PubMed, Scopus, and Web of Science were systematically searched for studies published from January 1, 2013, to December 30, 2024. A total of 58 cohort studies involving 7.5 million participants were ultimately included. Data on true positives, false positives, true negatives, and false negatives were extracted to compute pooled sensitivity, specificity, positive and negative likelihood ratios, diagnostic odds ratios (DOR), and area under the receiver operating characteristic curve (AUC). Summary receiver operating characteristic (SROC) curves were constructed, publication bias was evaluated using Deeks’ funnel plot asymmetry test, and clinical utility was assessed through Fagan nomograms. Analyses were performed in STATA version 18.

**Results:**

Across 58 studies, 47,276 CVD events occurred in men and 33,931 in women. Pooled sensitivity and specificity were 0.79 (95% CI: 0.77–0.89) and 0.66 (95% CI: 0.50–0.79) for the Framingham Risk Score (FRS); 0.74 (95% CI: 0.68–0.80) and 0.79 (95% CI: 0.43–0.95) for the ACC/AHA model; 0.77 (95% CI: 0.57–0.89) and 0.69 (95% CI: 0.43–0.89) for SCORE; and 0.85 (95% CI: 0.81–0.88) and 0.80 (95% CI: 0.79–0.81) for COX models. The highest pooled AUCs were observed for SCORE (0.81, 95% CI: 0.77–0.84) and COX (0.89, 95% CI: 0.89–0.92), indicating superior discrimination.

**Conclusions:**

Based on pooled AUCs, the most reliable threshold‐independent metric, SCORE and COX demonstrated superior performance, followed by FRS. While sensitivity/specificity and DOR provide classification insights, AUC offers the most robust basis for cross‐model comparison. Future research should focus on validating and comparing established models, adapting them locally, and integrating novel predictors, rather than developing redundant ones.

## 1 Introduction

Cardiovascular disease (CVD) is among the leading causes of mortality worldwide, accounting for approximately one‐third of all deaths [1]. Preventing CVD requires the timely identification of individuals at high risk to enable effective interventions, such as adjustments to diet, lifestyle, or pharmacological treatment [2]. Risk prediction tools are essential for the early identification, prevention, and treatment of individuals at high risk of a cardiovascular event (fatal or non‐fatal) and for targeting preventive strategies to those who will benefit most [3, 4]. The likelihood of heart disease is assessed based on known risk factors using composite scores. A large longitudinal study (Framingham), conducted in the United States since 1948, has provided evidence for the effects of risk factors incorporated into these scores [5].

A wide range of CVD risk prediction models have been developed by combining multiple risk factors over the past two decades. These include the Framingham Risk Score (FRS), the Australian Risk Score (ARS), the Pooled Cohort Equations (PCE), and the Systematic Coronary Risk Evaluation (SCORE‐high and SCORE‐low), which are used in developed countries, as well as the World Health Organization Risk Score (WHO‐RS) and Globorisk, which are common CVD risk prediction models in developing and less‐developed countries [4, 6].

Researchers have been developing and validating risk prediction models for decades to improve prediction accuracy. With technological advancements, development and validation methods have become more refined, efficient, and accurate, offering greater predictive capacity [7]. The predictive performance of older models is weaker compared to that of newer models. Newer technologies are constantly emerging, creating opportunities for better, innovative approaches and improved outcome prediction capabilities [8]. Therefore, older models need to be recalibrated and updated to account for changes in patient populations and their outcome risk profiles to function as effective risk prediction models [9].

There is a lack of well‐developed and validated risk prediction tools in low‐ and middle‐income countries; however, these risk prediction tools, to be used in resource‐limited settings, must be valid, practical, and cost‐effective in the specific environments in which they are applied [10]. Although numerous CVD risk prediction models exist, it is projected that annual cardiovascular disease mortality will increase from 17.5 million in 2012 to 22.2 million by 2030 [11]. This underscores the importance of CVD risk prediction models for public health interventions and stimulates the need for further improvements in existing models [12].

Numerous reviews have shown that prediction models exist for a wide range of CVD outcomes [13]. However, the most comprehensive reviews conducted over a decade ago (search up to 2013) indicate that the number of published models has significantly increased since then; furthermore, these reviews have not systematically described the outcomes that the models aim to predict, the most common predictors, the predictive performance of all models, and the models that have been externally validated [12, 14].

This systematic review and meta‐analysis addresses this gap by evaluating the diagnostic accuracy of CVD prediction models using prospective studies to minimize bias and provide robust evidence for clinical application. Specifically, we identify predicted outcomes, common predictors, and performance measures (sensitivity, specificity, and AUC), while assessing external validation. Our findings aim to strengthen early detection and prevention strategies, particularly in resource‐limited settings, and to inform cost‐effective public health interventions to curb rising CVD mortality.

## 2 Material and Methods

This systematic review and diagnostic meta‐analysis was prospectively registered in PROSPERO under identifier CRD420251124694 and adheres strictly to the PRISMA‐DTA 2020 guideline for diagnostic test accuracy studies [15].

### 2.1 Search Strategy

The literature search was carried out in PubMed, Scopus, and Web of Science to identify studies evaluating the diagnostic accuracy of cardiovascular disease (CVD) prediction models. Validated search filters tailored to study design were applied, enabling access to MEDLINE (via PubMed), Embase (via OVID), and CINAHL (via EBSCOhost) (Supporting File, Table S1). The search covered the period from July 1, 2013, to July 31, 2025, and was supplemented by earlier key studies identified through seminal reviews by Damen et al. [12] and Siontis et al. [16]. Reference lists of included articles were also screened to capture additional eligible studies.

### 2.2 Study Selection

Titles and abstracts were independently screened by two reviewers, followed by full‐text assessment of potentially relevant articles. Discrepancies were resolved through consensus or consultation with a third reviewer. The selection process was guided by the PIRT (Population, Index test, Reference test, Target condition) framework, with detailed criteria provided in Supporting File, Table S2.

### 2.3 Inclusion and Exclusion Criteria

Inclusion Criteria [1]: Published manuscripts that assess prediction models specifically developed for individuals across all age groups and geographic locations, with a focus on the primary prevention of cardiovascular disease (CVD) [2]; Acceptable studies encompassed both retrospective and prospective cohort analyses [3]; The published works clearly outlined the diagnostic standards for CVD [4]; Reports provided sensitivity, specificity, and optimal diagnostic cutoffs either directly or through data amenable to calculation.

Exclusion Criteria [1]: Systematic reviews, narrative reviews, case series, and individual case studies [2]; Manuscripts with insufficient information, particularly where critical metrics like sensitivity and specificity could not be derived [3]; Investigations without a well‐defined outcome measure or those restricted to models predicting risks solely for venous disease or isolated stroke [4]; Studies exploring the incremental benefit of additional predictors to existing models, those analyzing a single risk factor, or articles detailing models crafted or validated only within narrowly defined populations, such as individuals with diabetes, HIV, atrial fibrillation, or those subjected to cardiac interventions [5]; Literature that has been contested or retracted.

### 2.4 Data Extraction

Data extraction was performed independently by two reviewers (MAR and YM). The following study characteristics were recorded: first author, country, year of publication, prediction horizon, study design, study population, sample size (men/women), type of prediction model, outcomes, number of events, and diagnostic accuracy data (true positives (TP), false positives (FP), false negatives (FN), true negatives [17], sensitivity, specificity, area under the receiver operating characteristic curve (AUC), positive likelihood ratio (PLR), negative likelihood ratio (NLR), and diagnostic odds ratio (DOR)). One reviewer (MAR) conducted the full‐text review and extracted the data, while the second reviewer (YM) verified accuracy. Discrepancies were resolved through discussion with a third reviewer (GM). For studies with incomplete data, corresponding authors were contacted to obtain missing information.

### 2.5 Quality Assessment

The evaluation of study quality utilized the QUADAS‐2 (Quality Assessment of Diagnostic Accuracy Studies 2) framework, which examines potential bias across four key areas: patient selection, index test, reference standard, and flow and timing. However, given that QUADAS‐2 is not designed to assess predictive models for diagnostic or prognostic purposes [18], we supplemented this approach by incorporating the PROBAST (Prediction Model Risk of Bias Assessment Tool) [19], an instrument specifically tailored for evaluating predictive models with binary outcomes. Additionally, PROBAST analyzed bias risk in four distinct domains: participants, predictors, outcomes, and analytical methods.

### 2.6 Conceptual Justification and Limitations of Applying Diagnostic Accuracy Metrics to Prognostic Models

Although CVD risk prediction models are fundamentally prognostic tools intended to estimate future event probabilities rather than diagnose existing disease, many external validation studies operationalize them as binary classifiers by applying established clinical risk thresholds (e.g., ≥ 10% 10‐year risk) to stratify individuals for preventive therapy. This practice enables calculation of classification performance metrics (sensitivity, specificity, likelihood ratios, DOR) against observed binary outcomes, which are clinically relevant for guiding decisions such as statin initiation or intensified lifestyle intervention. Similar approaches have been adopted in meta‐analyses of prognostic models treated as classifiers in other fields, and are consistent with extensions of PRISMA‐DTA guidance for studies reporting threshold‐based performance.

Nevertheless, we recognize several important limitations. First, these metrics are inherently threshold‐dependent; different cut‐offs across studies (e.g., 7.5% for ACC/AHA PCE vs. 10% for FRS/SCORE) can substantially alter sensitivity and specificity. Second, pooling across heterogeneous thresholds may reduce interpretability and introduce bias. Third, diagnostic metrics do not capture key prognostic attributes such as calibration (agreement between predicted and observed risks) or time‐to‐event dynamics, which are central to model utility.

To mitigate these concerns, we tested for threshold effects using Spearman's rank correlation between sensitivity and (1 – specificity) and prioritized guideline‐recommended thresholds in data extraction. The bivariate random‐effects model was employed to jointly model the correlation between sensitivity and specificity, implicitly accounting for threshold‐related trade‐offs. Sensitivity analyses were conducted, restricting to the most common cut‐off (10%) where sufficient studies permitted. All pooled estimates of sensitivity/specificity/DOR should therefore be interpreted as threshold‐dependent classification performance summaries rather than absolute diagnostic accuracy.

### 2.7 Diagnostic Performance and Clinical Value Verification

An SROC (summary receiver operating characteristic) curve was constructed, and the corresponding area under the curve (AUC) was computed to assess diagnostic performance. To investigate publication bias, Deeks' funnel plot asymmetry test was employed, a preferred technique in diagnostic test accuracy meta‐analyses for examining the relationship between sample size and diagnostic odds ratio (DOR) [20]. A *p*‐value less than 0.05 was deemed suggestive of possible bias, aligning with Cochrane Handbook guidelines [21]. Additionally, a Fagan chart was created to determine pre‐test and post‐test probabilities, facilitating an evaluation of the clinical utility of the findings [22].

### 2.8 Bivariate Model Analysis

The presence of threshold effects was evaluated using Spearman's correlation coefficient, calculated between sensitivity and (1‐specificity), where a *P*‐value below 0.05 was taken as evidence of a notable threshold effect [23]. Furthermore, a bivariate random‐effects model was utilized to address the inherent correlation between sensitivity and specificity, delivering pooled estimates accompanied by 95% confidence regions [24].

### 2.9 Threshold Effects and Cut‐Off Harmonization

Threshold effects arise when studies use different risk cut‐offs to classify individuals as “high‐risk” or “low‐risk,” leading to an inverse relationship between sensitivity and specificity. To assess whether threshold variation was a major source of heterogeneity, we calculated Spearman's rank correlation coefficient (*ρ*) between sensitivity and (1 – specificity) for each major model. A statistically significant negative correlation (typically *ρ* < –0.5 and *p* < 0.05) indicates a notable threshold effect.

### 2.10 Heterogeneity Analysis

The degree of heterogeneity was assessed using the I² and Q statistical tests. An I² value exceeding 50% was interpreted as evidence of considerable heterogeneity, while a value surpassing 75% indicated severe heterogeneity. In cases where significant heterogeneity was detected, a random‐effects model was adopted; conversely, when heterogeneity was minimal, a fixed‐effects model was utilized [25]. Subgroup analysis and meta‐regression analysis were performed to explore the sources of heterogeneity. Variables included were predefined as follows: publication year (before 2015 vs. 2020–2025), region (USA vs other countries), outcome (fatal and non‐fatal), and prediction horizons (10 years vs 5 years).

### 2.11 Statistical Analysis

All statistical evaluations were conducted using STATA version 18.0. The I² statistic was employed to measure heterogeneity, with a fixed‐effects model implemented when heterogeneity was low (I² < 50%), and a random‐effects model adopted otherwise. Sensitivity was calculated as TP/(TP + FN), and specificity as TN/(TN + FP). PLR, NLR, and DOR were computed. SROC curves and AUC were generated. Publication bias was evaluated through the use of a funnel plot and Deeks' test. Additionally, regression analysis was performed to investigate the influence of covariates such as model type, population size, and input features on diagnostic accuracy.

## 3 Results

### 3.1 Literature Search

**Figure 1:**
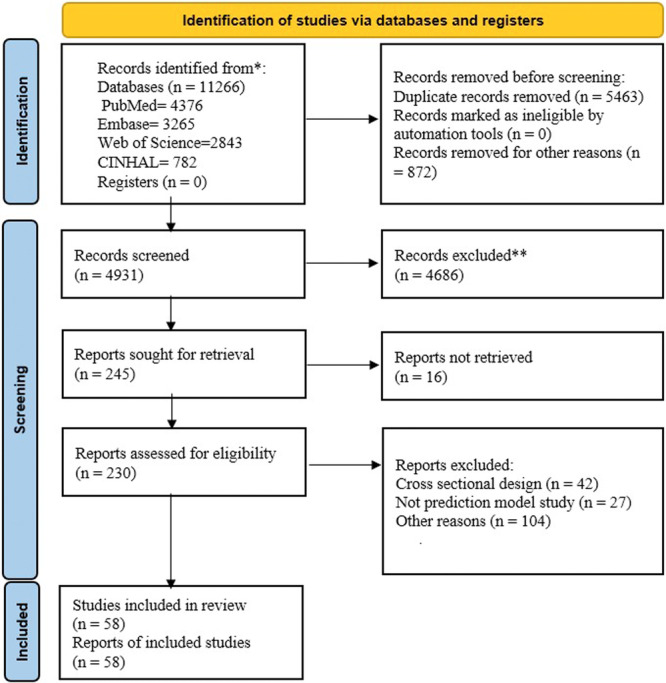
PRISMA diagram of indicated studies.

The initial search yielded 11,266 results. After removing duplicates, 4931 articles remained and were screened based on their titles and abstracts. Of these, 4686 articles were excluded due to irrelevance, and 245 articles underwent full‐text screening. Finally, 58 cohort studies met all eligibility criteria and were included in the review. Using the PRISMA flow diagram as a guide, the processes for identifying, screening, and including or excluding records were conducted (Figure 1).

### 3.2 Basic Characteristics

Table 1 summarizes all included studies. This meta‐analysis incorporated 58 studies investigating the diagnostic accuracy of CVD prediction models. The studies spanned from 2013 to 2025. Prediction horizons varied, with the majority focusing on 10‐year risk (33 studies), followed by 5‐year risk (10 studies), and other durations including 4 years, 18.7 years, lifetime, and mixed follow‐ups (e.g., 5–10 years, 10.2–16.4 years). All studies were prospective or retrospective cohort studies. Study populations were diverse, covering multiple geographical regions such as Europe (e.g., Netherlands, Germany, UK), North America (e.g., USA), Asia (e.g., China, South Korea, Japan), Australia, and the Middle East (e.g., UAE).

**Table 1:** Characteristics of the included studies.

ID	Author (Country)	Year	Prediction horizon (years)	Study Design	Study population	Sample size (men/women)	Models	Outcomes	No of events (men/women)
1	Kist, Netherlands [26]	2023	10	Prospective, dynamic, population‐based cohort	Individuals aged 40–70, no prior CVD/diabetes	Total = 155,013; Men = 74,880; Women = 80,133	SCORE2 CVD risk models	First CVD event (stroke, MI, CVD death)	Men = 4251; Women = 2715; Total = 6966
2	Angelow, Germany [27]	2022	10	Longitudinal cohort with inverse probability weighting	No prior CVD, aged 30–80, from the SHIP cohort	Total: 196 (9.8%); Men/Women not split; inferred higher in men	Arriba instrument (online version)	10‐year fatal/non‐fatal CVD events	Total: 196 (9.8%); M/W not split; inferred higher in men
3	Hageman, Multi‐country (UK, USA, Netherlands, Germany) [28]	2023	10	Prospective cohort	Apparently healthy, no prior CVD/DM	Derivation: 409,757 (sex not split); CPRD: 518,015	SCORE2 + risk modifiers	10‐year CVD events (fatal/non‐fatal); non‐CVD death as competing risk	Derivation: 16,166 CVD, 19,149 non‐CVD; CPRD: 12,675 CVD, 28,998 non‐CVD (sex not split)
4	Pylypchuk, New Zealand [4]	2018	5	Prospective open cohort study	Primary care patients aged 30–74, no prior CVD/renal/heart failure	Men: 198,839 (49.5%); Women: 202,913 (50.5%) (total: 401,752)	PREDICT‐1st equations, PCEs	Total CVD (hospitalization/death: IHD, stroke, PVD, CHF, etc.)	Total: 15,386 (Men: 8560, 55.6%; Women: 6826, 44.4%, inferred)
5	Backholer, Australia [29]	2017	5	Prospective cohort study (pooled data)	General population aged 40–74, no prior CVD, from 6 Australian cohorts	Men: 22,466 (41%); Women: 32,363 (59%) (total: 54,829)	Australian 5‐year CVD mortality risk score (primary, low‐info, traditional)	5‐year CVD mortality (CHD, cerebrovascular disease)	Total: 1375 (Men: 564, 41%; Women: 811, 59%, inferred)
6	Artigao‐Rodenas, Spain [30]	2013	10	Population‐based cohort study	General population aged 30–74, no prior CVD	Men: 339 (44.7%); Women: 420 (55.3%) (total: 759)	Framingham Risk Score (D'Agostino reclassification model)	10‐year CVD incidence (CHD, CVE, PAD, lethal HF)	Men: 43 (12.7%); Women: 31 (7.4%) (total: 74, 9.7%)
7	Al‐Shamsi, UAE [31]	2020	10	Retrospective cohort study	UAE nationals aged 30–79, no CVD or diabetes at baseline	Men: 249 (44.9%); Women: 305 (55.1%) (total: 554)	Framingham Risk Score (FRS)	10‐year hCHD incidence (myocardial infarction, coronary death)	Men: NR; Women: NR (total: 26, 4.7%)
8	Shan, China [32]	2022	4	Retrospective cohort study	Chinese males aged 30–74, no CVD at baseline	Men: 2331; Women: 0	CVDMCM (Model 2), Framingham CVD, Wu's Simplified Model	4‐year CVD incidence (CHD, ischemic stroke)	Men: 130; Women: N/A (total: 130)
9	Qiu, China [33]	2023	10	Retrospective cohort study	Rural adults 25–74, no CHD (China)	570 men, 811 women	FRS, China‐PAR	10‐year CHD incidence	Men: 69 Women: 91
10	Lindbohm, United Kingdom [34]	2021	18.7	Longitudinal cohort with follow‐up	Civil servants 40–75, no prior CVD (UK)	5233 men, 2341 women	SCORE, ASCVD	Incident CVD, CVD‐free life‐years	Men: 1042; Women: 399
11	Lim, South Korea [35]	2024	10	Retrospective cohort study	Adults > 30 years	84,087 50,619 men, 33,468 women (total); FRS: 50,606 men, 26,790 women; ASCVD: 33,158 men, 25,146 women	Framingham Risk Score (FRS), ASCVD Risk Score, Data‐driven model	10‐year incidence of CVD (ischemic heart disease, coronary heart disease, stroke, etc.)	Men: 3294 total (e.g., 1203 cerebral infarction); Women: 2478 total (e.g., 1078 cerebral infarction)
12	Stenling, Sweeden [36]	2020	10	Retrospective cohort	Middle‐aged adults [28, 37, 38]	Total: 92,915 (Men: 45,400; Women: 47,515)	Gender‐ and age‐specific Gompertz models	Non‐fatal CHD, stroke, HF, CVD death, non‐CVD death	Total: 13,247 (CHD: 5532; Stroke: 2269; HF: 518; Non‐CVD: 3841; CVD: 1087)
13	Hsu, Taiwan [39]	2024	5	Retrospective cohort	Asians ≥ 75 years, no prior CVD	Total: 12,174 (Derivation: Men 4358, Women 5382)	Sex‐specific Cox models	MACE (non‐fatal MI, ischemic stroke, CV death)	Men 590, Women 608
14	Chun, China [37]	2018	9	Prospective cohort study	Adults aged 30–79 years	Total: 503,842: Men: 205,293 (40.7%, Women: 298,549 (59%)	FSRP 2017	Total stroke	Total Stroke: 43,234 (Men: 19,587, Women: 23,647)
								Ischemic stroke	Ischemic Stroke (IS): 36,310 (Men: 16,113.
								Hemorrhagic stroke	Women: 20,197, Hemorrhagic Stroke (HS): 8865 (Men: 4587, Women: 4278)
15	Shahlan Kasim, Malaysia [40]	2023	10	Cohort prospective	Adults aged 18–92 years	12573 FRS: 10,145 (men: 4318, women: 5827).	Framingham Risk Score (FRS), SCORE2, Revised Pooled Cohort Equations (RPCE), WHO CVD	CVD: Myocardial infarction (acute/silent), coronary surgery, stroke, atherosclerosis, stent implants.	WHO CVD: 3.4% of 9265 ≈ 315 events.
						SCORE2: 7423 (men: 3157, women: 4266).		Ascertainment: Medical records (CVD) and survey (non‐CVD)	RPCE: 4.6% of 9162 ≈ 421 events.
						RPCE: 9162 (men: 3989, women: 5173).			FRS and SCORE2 event counts not specified (minimum 100 events per group)
						WHO‐CVD: 9265 (sex split not specified).			
16	Hosein, Trinidad and Tobago [41]	2019	10	Cohort	Adults aged 18–75 years	778 (Men: 368 /Women: 410)	ASSIGN Risk Score, Framingham Risk Score, QRISK2 Risk Score	Known CVD status (presence or absence of a prior CVD event)	Total: 252 CVD participants: 143 men, 110 women
17	Mansoor, USA [42]	2019	10	Cohort prospective	Adults aged 45–64 years from ARIC, no prior CVD at baseline	9285 (Men: 3987; Women: 5298)	EZ‐CVD (6 self‐reported items); ASCVD (lab‐based comparator)	Incident CVD events (CHD, stroke, mortality)	Total: 694 (7.47%); Men/Women breakdown not specified
18	Yang, China [43]	2016	10	Cohort	Derivation cohort: two cohorts) The China‐PAR project used InterASIA and China MUCA (1998) to develop the Chinese ASCVD risk equations Validation cohort from two independent cohorts, MUCA (1992–1994) and CIMIC, with 14,123 and 70,838 participants.	21,320, 35–74 years old	The China‐PAR Project compared with the PCE	Incident ASCVDevents	Derivation cohort: 21,320; average follow‐up of 12.3 years, 1048 incident ASCVD events (645 in men and 403 in women) were identified
19	Veronesi, Italy [44]	2017	16	Cohort	Data from seven cohorts recruited between 1986 and 1996 in Brianza and in Latina (the MATISS study). All cohorts took part in the collaborative CUORE Project	8328 (men: 3935, women: 4393)	“combined” (SCORE R CAMUNI‐ MATISS) vs “current” SCORE alone stratification	Major CVD event, fatal or nonfatal	CVD events: 468 in men and 210 in women
20	Van Staa, UK [45]	2014	10	HER cohort	Using the November 2011 version of CPRD and drawn from CPRD practices in the UK GP that participated in the linkages	1.8 million aged 35–74 years	FRS ASSIGN QRISK2	CVD as recorded by the GPs (MI, angina, CHD, stroke, and transient ischemic attack). Hospitalization due to CVD Death due to CVD	CVD outcomes occurred in 69,870 persons
21	Tralhao, Portugal [46]	2016	N/A	Cohort	Single‐center prospective registry of patients undergoing coronary computed tomography angiography, aged 40–75 years without diabetes or known cardiovascular disease.	327 patients assessed (181 men,146 women), mean age 59 ± 9 years	PCE SCORE	High coronary Atherosclerosis burden; coronary artery calcium score (CACS)	45% no visible coronary calcification; only 27 (8%) had a CACS greater than 400. CACS was quantified by the Agatston method, and a CACS cutoff value of greater than or equal to 300 was used to define severe coronary artery calcification
22	Tillin, UK [47]	2014	Baseline 1988–1991 Follow‐up 2008–2011	Cohort prospective	SABRE 1866 white Europeans, 1377 South Asians, and 578 African Caribbeans,	4539 at baseline, 4228 at follow‐up, 3821 had follow‐up data, aged 40–69 at baseline, men and women	QRISK2 versus Framingham 3 with South Asian adjustment	First CVD events: myocardial infarction, coronary revascularization, angina, transient ischemic attack or stroke reported by participant, primary care or hospital records, or death certificate records or death	Follow‐up data were available for 3821 (90%). 387 (14%) CVD events occurred in men and 78 in women (8%); 82% of these were CHD events 82% of these were CHD events
23	Sussman, USA [48]	2017	5	Cohort	VA ambulatory care services HER data: aged 45–80 years old (mean 61.7 ± 8.6); 95% male at specific ambulatory care clinics in 2006.	1,512,092 patients (1,435,937 men; 76,155 men)	recalibrated VA Risk Score‐CVD (VARS‐CVD) and ASCVD score. Population Recalibrated, Regression Recalibrated	Hard CVD events: the first occurrence of nonfatal myocardial infarction, CHD death, fatal or nonfatal stroke	Not reported
24	Sun, China [38]	2017	6	Cohort	Chinese rural population of Henan Province, China aged 40–65 years	10,338; male: 3945 (38.16) female: 6393 (61.84)	General‐FRS, Simplified FRS, SCORE‐high, SCORE‐low, CN‐ ICVD, PCE‐white and PCE‐AA were assessed	CVD deaths	168 CVD deaths
25	Selvarajah, Malaysia [7]	2014	5	Cohort	The 2006 National Health and Morbidity Survey dataset; 14,863 participants aged 40–60 years (mean 50.4 [7])	14,863 participants aged 40–60 years (mean 50.4), male: 45.3%, women: 54.7%	The FRS, SCORE WHO/ISH models	CVD mortality	98 events in men, 50 events in women
26	Sarrafzadegan, Iran [49]	2017	10	Cohort	The Isfahan Cohort Study (ICS)	5,432 participants (Average age for men and women was 51.2 ± 11.9 and 50.3 ± 11.3 years, respectively; 2784 women, 51.3%)	Gender‐specific PARS risk chart compared with the Framingham model	The risk of ischemic CVD events, including sudden cardiac death due to unstable angina, myocardial infarction, and stroke	705 events (564 IHDs, 141 strokes)
27	Qureshi, USA [50]	2016	10	Cohort	MESA; 47.1% men; 37.1% whites; 27.2% blacks; 22.3% Hispanics; 12.0% Chinese Americans	5,654 mean age 61.4 years, 47% male, and 53% female	PCE criterion Modified FRS SCORE	Incident ASCVD, composed of fatal and nonfatal myocardial infarction, other fatal and nonfatal coronary heart disease, fatal and nonfatal cerebrovascular disease, and fatal/ nonfatal other atherosclerotic	642 (6%) incident AVCD events
28	Pursnani, USA [51]	2015	9.4	Cohort	Third generation cohorts of the Framingham Heart Study; Framingham multidetector computed tomography (MDCT) imaging study (2002–2005)	2435 participants, mean age 51.3 (SD, 8.6), 56 women, and the mean FRS 6.7%	The ACC/AHA guidelines versus the National Cholesterol Education Program's 2004 Updated Third Report of the Expert Panel on Detection, Evaluation, and Treatment of High Blood Cholesterol in Adults (ATP III) guidelines	disease deaths; The primary outcome was incident CVD (MI, death due to CHD, or ischemic stroke). Secondary outcomes were CHD and CAC (as measured by the Agatston score)	74 incident CVD events (40 nonfatal myocardial infarctions, 31 nonfatal ischemic strokes, and 3 fatal CHD events)
29	Pylypchuk, New Zealand [4]	2018	4.2	Cohort prospective	Primary Care; 401,752 people between Aug 27, and Oct 12, 2015, European, Indian, Maori, Chinese, or other Asian	age 54.5 ± 12.9. 401,752 226,053 men and 175,699 women aged 30–74 years (mean age (SD) women = 56 (8.9); Men = 51.8 (9.9)	New PREDICT absolute risk prediction equations versus ACC/AHA Pooled‐Cohort Equations	IHD (including angina); ischemic or hemorrhagic cerebrovascular events (including transient ischemic attacks); or peripheral vascular disease, congestive heart failure, or other ischemic cardiovascular	15,386 (4%) participants had CVD events (1507 [10%] were fatal, and 8549 [56%] met the PCEs definition of hard ASCVD during 1,685,521 person–years follow‐up.
30	Nishimura, Japan [52]	2014	11.8	Cohort	Urban population: The Suita cohort study started in 1989	5521 (male 2796; mean age 56.1 ± 13.3 and female 2725; mean	The Suita Score compared with the FRS (the original and the calibrated)	The incidence of CHD, MI, and sudden cardiac death	213 cases of CHD
31	Mortensen, Denmark [53]	2017	5–10	Cohort	44,889 individuals aged 40–75 recruited in 2003–2009 in the Copenhagen General Population Study; all free of ASCVD, diabetes, and statin use at baseline	44,889 men =19,383 women ±25,506	USPCE for any ASCVD European‐ SCORE for fatal ASCVD	Comparison of ACC/AHA guidelines to the ESC/EAS guidelines for the primary prevention of ASCVD, for accurately assigning statin therapy	2217 any ASCVD events and 199 fatal ASCVD events through 2014
32	Mortensen, Denmark [54]	2015	10	Cohort	CGPS (Copenhagen General Population Study): (48–64 years);	37,892 57% women	Risk‐based approach – ACC/ AHA Guidelines versus Trial – based approach – Ridker et al. versus Hybrid approach – Ridker et al.	ASCVD events, MI	ASCVD events= 834; MI= 323
33	Marrugat, Spain [55]	2014	10	Cohort	The FRESCO study (Función de Riesgo ESpañola de acontecimientos Coronarios y Otros, or “Spanish risk function of coronary and other cardiovascular events”), 11 population cohorts in 7 Spanish regions, examined between 1992 and 2005:	50,408 participants in total, aged between 35 and 79 years, 23,289 (46.2%) men and 27,119 (53.8%) women	A set of 10‐year cardiovascular risk predictive functions in Spain versus Framingham‐ REGIGOR	CV events (fatal and nonfatal CHD, stroke, fatal events, MI, angina, other CV)	2973 cardiovascular events Fatal events (*n* = 2301) at least one nonfatal CV event (*n* = 2500)
34	Lee, Hong Kong [56]	2015	10	Cohort	The Hong Kong Cardiovascular Risk Factor Prevalence Study (CRISPS): aged 25–74 years	2895 Chinese men and women	PCE versus FRS CV risk estimation	CV event, CHD, stroke, all fatal and nonfatal events (MI)	PCE of 1476 subjects aged 40–79, 122 developed hard ASCVD (80 men, 42 women). The Framingham CV risk
35	Lee, Korea [57]	2014	10	Cohort	Korea population	KNHANES: 7594 Korean adults, aged 40–79 years	7594 male =3307, Women =4287	PCE versus ATP‐III equation	ASCVD risk and CHD risk; also, lipid management eligibility
36	Kempf, Germany [58]	2016	10	Cohort	The Boehringer Ingelheim cohort 4005 participants; mean age 46.7 ± 5.8; men (54%), 134 men (6%), and 111 women (6%) had current CVD	4005	FRS, PRS, RRS	10‐year CVD risk estimation	10‐year CVD risks of 35% (FRS), 9% (PRS), and 6% (RRS) for men and 10% (FRS), 4% (PRS), and 1% (RRS) for women among 1668 subjects aged 30–74 years, 138 developed CVD (86 men, 52 women)
37	Kavousi, Netherlands [59]	2014	10	Cohort	Rotterdam Study participants aged 55 years or older in the Ommoord district of Rotterdam.	4,854 aged 55 years or older, the mean age of participants was 65.5(SD, 5.2) years, and 54.5% were women.	Guidelines: ACC/ AHA; ATP‐III; and ESC	“hard” ASCVD events (including fatal and nonfatal CHD and stroke) (ACC/AHA), hard CHD events (fatal and nonfatal MI, CHD mortality) (ATP‐III), and the ASCVD mortality (ESC).	1. ACC/AHA: hard ASCVD 343; men = 192; women = 151 2. ATP‐III: 160; men = 98; women = 62; 3. ESC: CVD death = 87 (men = 50; women = 37
38	Karjalainen, Sweden [60]	2017	10	Cohort	The Northern Sweden MONICA 2014 cohort: 40– 65 years	813, 53% women	2003 SCORE Sweden vs. 2015 SCORE Sweden	The high and very high‐risk groups for Cardiovascular death	2015, SCORE Sweden observed CVD death 34 vs. predicted 44.3. In 2003, SCORE Sweden observed CVD death 34 vs predicted 77.3
39	Kariuki, USA [61]	2017	12	Cohort	Atherosclerosis Risk in Communities (ARIC) dataset: Secondary analysis. 11,601 participants (mean age, 54 years; 23% black)	11,601 55% women	The nonlab FRS algorithm versus the lab‐based FRS algorithm	CVD events (coronary death, myocardial infarction, coronary artery bypass grafting, and percutaneous Coronary intervention), cerebrovascular events (i.e., ischemic stroke, hemorrhagic stroke, and transient ischemic attack), and heart failure	1545 new cases of CVD
40	Jung, Korea [62]	2015	10	Cohort	The KHS (the Korean Heart Study): aged 40– 79 years (1996–2001); Men =119,715 (mean age 50.13 ± 7.94); (mean age 51.81 ± 8.12)	200,010 adults 80,293 women 119,717 men	PCE versus a KRPM	First “hard” ASCVD events, comprising the occurrence of death from CHD or fatal stroke, or the first occurrence of nonfatal MI or stroke	12,327 ASCVD events (2175 nonfatal MI, 478 fatal CHD, 10,049 nonfatal stroke, and 749 fatal stroke)
41	Johansson, Finland [63]	2016	11.2	Cohort	The Health 2000 Study from Finland between autumn 2000 and spring 2001; mean age 51.9 ± 14.5	5843 44.8% men	CHD risk score Health 2000; Finrisk, Framingham, and RRS coronary artery bypass surgery	Cardiovascular mortality, nonfatal myocardial infarction, nonfatal stroke, and percutaneous coronary intervention	CV event = 557 No CV event = 5286
42	Jee, Korea [64]	2014	11.6	Cohort Prospective	The Korean Heart Study (KHS)	268,315 164,005 men, 104,310 women Koreans aged 30–74 years	Develop a CHD risk model among the Korean Heart Study (KHS) population and compare it with the Framingham	Nonfatal or fatal CHD events between 1997 and 2011.	2596 CHD events (1903 nonfatal (MI) and 693 fatal (e.g., sudden death)
43	Hua, Australia [65]	2017	5 and 10 years, Maximal follow‐up time of 10.5 and 16.4 years	Cohort	The Well Person's Health Check Indigenous cohort 1448 (women = 748; mean age 45.2 ± 11.6; men = 700, mean age 44.9 ± 11.0) Aboriginal and Torres Strait Islanders from remote Indigenous communities in Far North Queensland.	1448 (748 women; 700 men)	FRS 1991; FRS 2008; Recalibrated FRS 2008	CHD (including myocardial infarction, angina Pectoris, and coronary insufficiency), CHD death, stroke, congestive heart failure, and peripheral vascular disease	369 CVD events (25.5%)
44	Hu, USA [66]	2014	ARIC 10 years NHANES 14 years	Cohort	1. ARIC (the Atherosclerosis Risk in Communities): 13,657 participants, aged 45–64 years 2. NHANES III *n* = 5706; 40–70 years old individuals	19,363 males and females	1. FRSv1from 1998 2. FRSv2 from National Cholesterol Education Program Adult Treatment Panel III, 3. FRSHDL in which the high‐density lipoprotein (HDL) component of FRSv1 was ignored 4. NEW CHD with a new‐CHD model	MI, fatal CHD, CHD‐ associated death	ARIC = 13,657; 759 CHD cases, NHANES III *n* = 5706; 88 CHD‐associated deaths
45	Harari, Israel [67]	2017	10 and 20	Cohort	A longitudinal Israeli industrial cohort (CORDIS cohort)	4809 all male	FHS SCORE modified FHS model (FHS/Cox) Omnibus/Cox	CV mortality	76 CVD mortality events during the 10‐year follow‐up period and 170 during the 20‐year follow‐up period
46	Goh, Australia [68]	2014 a	10	Cohort	The National Heart Foundation Third Risk Factor Prevalence Study: 4487	4,487 Australian women; age between 20 –69 years; Mean 42.8 ± 13.2 years	FRS SCORE risk charts for low‐risk regions, SCORE risk charts for high‐risk regions in Europe	claudication Death (all cause); CVD deaths	death (all cause) = 152; CVD deaths = 28
47	Goh, Australia [69]	2014 b	10	Cohort	The third Australian Risk Factor Prevalence Study (north and south Sydney, Melbourne, Brisbane, Adelaide, Perth, Hobart, Darwin, and Canberra)	4354 females aged 20–69 years	the FRS model versus SCORE	CHD and CVD incidence and/or mortality	Not reported
48	Fox, USA [70]	2016	10	Cohort	The Jackson Heart Study (JHS) External validated cohorts ARIC and MESA	3689 participants	ACC/AHA CVD risk algorithm versus FRS	Incident CVD event was defined as the first occurrence of myocardial infarction, coronary heart disease death, congestive heart failure, stroke, incident angina, or intermittent	270 CVD events (166 women)
49	Foraker, USA [71]	2016	10	Cohort	The Jackson Heart Study; *n* =4140. 5301 participants aged 21–95 years (median 54.5; 65% females) between 2000 and 2004, without prior history of stroke (5301); missing data (927); finally included (4140)	4140	CVD risk score versus CVH metric (the American Heart Association/ American Stroke Association)	The cumulative incidence of stroke, HR, and 95% CIs for stroke within African Americans	
50	Flueckier, USA [72]	2018	10.7	Cohort	MESA (Multi‐Ethnic Study of Atherosclerosis). The mean age was 62 ± 10 years. Thirteen percent had diabetes, 15% were on statin therapy, 20% on aspirin therapy, and 33% on antihypertensives.	6712, 53% female	The revised (R‐ FSRS), original FSRS, and the PCE	Stroke prediction. Stroke was defined as fatal/nonfatal strokes (hemorrhagic or ischemic).	231 stroke events [182 hemorrhagic (3.4%); 49 ischemic strokes (2.7%)]
51	Fatema, Bangladsh [73]	2016	2.5	Cohort	Rural Bangladeshi population. The original cohort was initiated in 2008 ‐ the “BADAS‐ORBIS Eye Care Project,” a rural Bangladeshi population (52989 cohort and 439 subcohort participants) mean age 53.73 ± 10.71 years	52,989 followed up until Aug 2014–Dec 2014 (participation rate, 85.02%) 439 followed up until Aug 2014–Dec 2014 (participation rate, 77.97%)	Model 1 FRS (Laboratory‐based) Model 2 (WHO with cholesterol) Model 3 (WHO without cholesterol) Model 4 FRS (Non‐laboratory based)	CHD (e.g., MI evidenced from ECG, CV death)	Disease free = 394; total cases = 60 (MI)
52	Dufouil, USA [74]	2017	5 and 10	Cohort	FHS, REGARDS, and 3C cohorts 1. the FHS: the old FSRP (*n* = 5734, age = 55); the contemporary epoch (*n* = 5.072, age = 55) 2. REGARDS (the REasons for Geographic and Ethnic Differences in Stroke) 30,239 participants = 45 years of age; 42% black and 58% white for 5‐year follow‐up. 3. the 3C study: three French cities (Bordeaux, Dijon, and Montpellier) *n* = 7601, at least 65 years old	43,574 45% men and 55% women	FSRP old FSRP new	all‐stroke and ischemic stroke	In the new FHS, over 10 years of follow‐up, there were a total of 277 incident strokes (247 ischemic strokes (IS)), 144 (129 IS) in women.
53	De las Heras Gala, Germany [75]	2016	10	Cohort	The Monitoring of Trends and Determinants in Cardiovascular Disease (MONICA)/ Cooperative Health Research in the Region of Augsburg (KORA) and the Heinz Nixdorf Recall (HNR) Studies. The KORA S3 (1994–1995) and S4 (1999–2001), total =5238; men, 49%; age 40–79 years old HNR =4208	9446, 48% men	the ACC/AHA risk score versus the ESC SCORE	nonfatal or fatal ASCVD events	KORA S3 and S4 = 383 (7.8%) incident of ASCVD events HNR =271 (6.4%) first incident ASCVD events
54	DeGoma, USA [76]	2013	10	Cohort	1. Framingham (Wilson et al., 1998) (mean age 49; women 53%; white 100%) 2. Women's Health Study (Ridker et al., 2007) (mean age 52; women 100%; white 95%) 3. Physician's Health Study (Ridker et al., 2008b)(mean age 63; women 0%; white N/A) 4. ARIC (Nambi et al., 2010) (mean age 54; women 57%; white 75%) 5. MESA (Goff et al., 2006)(mean age 62; women 53%; white 38%)	1. FRS (Wilson et al., 1998) (*n* = 5345) 2. Women's Health Study (Ridker et al., 2007) (*n* =16,400) 3. Physician's Health Study (Ridker et al., 2008b) (*n* = 10,724) 4. ARIC (Nambi et al., 2010) (*n* = 13,145) 5. MESA (Goff et al., 2006) (*n* = 6704)	FRS risk score, equations derived from the Multi‐ Ethnic Study of Atherosclerosis (MESA) and the Atherosclerosis Risk in Communities (ARIC) study, and the Reynolds risk score	Differences in absolute 10‐year cardiovascular risk between alternative risk equations. Absolute 10‐year cardiovascular risk of having CHD for Framingham, MESA, and ARIC risk equations. The Reynolds score includes CHD events, additionally non‐CHD death, coronary revascularization, and ischemic stroke. Differences in absolute risk of CV events.	Not reported
55	DeFilippis, USA [77]	2015	10.2	Cohort	MESA (Multi Ethnic Study of Atherosclerosis), a community‐based, sex‐balanced, multiethnic cohort (mean age, 61.5 years; women, 53.5%; white, 42%; African American, 26%; Hispanic, 20%; and Chinese, 12%)	4227 (1961 men, 2266 women)	Five risk scores: 1. FRS‐CHD 2. FRS‐CVD 3. ATP‐FRS‐CHD 4. RRS 5. AHA/ACC/ ASCVD	Observed and expected ASCVD events	Not reported
56	Chia, Malaysia [78]	2014	10	Retrospective cohort	Asian population: Chinese, Indian, and Malay (mean age: 57.5 ± 8.8 years)	922 66.7% female	PCE FRS General Cardiovascular Disease (CVD) risk score.	nonfatal myocardial infarction, coronary heart disease (CHD) death, fatal and nonfatal stroke	CVD events occurred in 45 of 922 patients, 22 (7.2%) in males and 23 (3.7%) in females
57	Boateng, Netherlands [79]	2018	10	Cohort	3586 participants aged 40–70 years in the multicenter RODAM (Research on Obesity and Diabetes among African Migrants) study among Ghanaians residing in Ghana and Europe	3586 All male (Ghana = 1564; Europe = 2022)	FRS lab FRS nonlab PCE algorithms.	Not specified; 10‐year CVD risk. Participants were classified as low, moderate, or high risk, corresponding to,10%, 10–20%, and 0.20%, respectively	Not reported
58	Bazo‐Alvares, Peru [80]	2015	10	Cohort	Peru: sites: Ayacucho (*n* = 83); Lima (*n* = 871); Puno (Rural) (*n* =356); Puno (Urban) (*n* = 366); and Tumbes (*n* = 495), age 45–54 (52.9%) and 55–65 (47.4%)	2183 48.1% male; 51.9% female;	PCE was compared with six other models: 1. FRS, Global CVD: two versions used 2. ACC/AHA model 3. WHO Risk Chart (WHO/ISH) 4. RRS 5. SCORE project risk score, 6. Risk Chart developed	in agreement with the predicted CVD risk using Lin's concordance correlation coefficient	Not reported

Abbreviations: ASSIGN, assessing cardiovascular risk to Scottish Intercollegiate Guidelines Network to assign preventative treatment; CABG, coronary artery bypass graft; China MUCA (1998), China Multi Center Collaborative Study of Cardiovascular Epidemiology; China‐PAR project, Prediction for ASCVD Risk in China; CN‐ICVD, Chinese ischemic CVD; CI, confidence intervals; CPRD, clinical practice research datalink; CIMIC, Community Intervention of Metabolic Syndrome in China & Chinese Family Health Study; CN‐ICVD, Chinese ischemic CVD; CVD, Cardiovascular disease; EHR, Electronic Health Record; ESC SCORE, European Society of Cardiology risk score; FRS, Framingham Risk Score; FRSP old, Framingham Stroke Risk Profile; FRSP new, Framingham Stroke Risk Profile new; gen‐FRS, general Framingham risk score; GFRS, Global Framingham risk score; sim‐FRS, simplified Framingham risk score; IHD, ischemic heart disease; Inter ASIA, International Collaborative Study of Cardiovascular Disease in Asia; KRPM, Korean Risk Prediction Model; LCD, lancet chronic disease; MATISS, Malattia Aterosclerotica Istituto Superiore di Sanità study; MESA, Multi‐Ethnic Study of Atherosclerosis; MI, myocardial infarction; MONICA‐Brianza and the PAMELA, Pressioni Arteriose Monitor e Loro Association studies; NA, not applicable; NI, Northern Ireland cohort.; PCE, Pooled‐ Cohort Equation [atherosclerotic cardiovascular disease (ASCVD) score]; PCE‐AA, Pooled‐Cohort Equations African American; PCE‐white, Pooled‐Cohort Equations White; PROCAM, prospective cardiovascular Münster score; PTCA, percutaneous transluminal coronary angioplasty; SABRE, Southall And Brent REvisited cohort; SCORE, systematic coronary risk evaluation; SCORE‐high, High‐risk systematic coronary risk evaluation; SCORE‐low, Low‐risk systematic coronary risk evaluation; Suita Score, coronary prediction algorithms for Japanese; VA, Veterans Affairs; WHO/ISH, WHO/International Society of Hypertension.

The total sample size across all studies exceeded 7.5 million participants, with sex distributions varying: 24 studies reported higher male participation, 16 had more females, and 18 did not specify sex splits or had balanced representation. Commonly used models included the FRS, ACC/AHA, PCE, SCORE, and various region‐specific models (e.g., China‐PAR, PREDICT‐1st). Outcomes primarily focused on incident CVD events, including myocardial infarction, stroke, CVD death, and composite endpoints, with some studies accounting for competing risks (e.g., non‐CVD death) (Table 1).

### 3.3 Quality Assessment Results

The evaluation of risk of bias using the QUADAS‐2 tool indicated an overall low risk of bias across the studies; however, approximately 27% of the investigations displayed significant bias related to patient selection (Supporting File, Table S3 and Figure S1).

### 3.4 PROBAST Checklist

The outcomes of the PROBAST quality assessment are presented in Supporting File Table S4. This evaluation scrutinized the quality of 47 prediction modeling studies related to CVD using the PROBAST checklist. The review concentrated on four key areas: participant selection, predictors, outcome, and analysis, while also considering applicability across these domains. Overall risk of bias and concerns regarding applicability were assessed. A low risk (+) was attributed to all 45 studies collectively, despite differing risk levels within individual domains, suggesting that strong analytical methods compensated for specific domain‐related issues. High applicability (+) was observed in 44 studies, whereas 3 studies, the Korean Heart Study, Korean Risk Prediction Model, and VARS CV, were rated with low (−) applicability due to issues in participant selection and predictors (Supporting File, Table S4).

### 3.5 Diagnostic Accuracy of Prediction Models for CVD

Pooled diagnostic parameters were computed using a random‐effects model (REM). The analysis featured forest plots illustrating the AUC for various CVD prediction models, alongside measures of sensitivity, specificity, PLR, NLR, DOR, and SROC curves. Pooled AUCs could not be generated for PCE and ASCVD due to insufficient studies (*n* < 4) providing complete ROC data or variance estimates, precluding stable meta‐analysis. This reflects a common reporting gap in external validations. Sex‐stratified AUCs are presented where available (Table 2).

**Table 2:** Results of subgroup analysis.

Models	Categories	Sensitivity	Specificity	AUC	PLR	NLR	DOR
**FRS**	Overall	0.79 (0.66–0.89)	0.66 (0.50–0.79)	0.77 (0.73–0.80)	2.3 (1.8–2.9)	0.40 (0.30–0.52)	6 (4–9)
	Man	0.62 (0.55–0.69)	0.75 (0.70–0.79)	0.74 (0.70–0.78)	2.3 (2–2.7)	0.51 (0.44–0.60)	5 (3–6)
	Woman	0.64 (0.56–0.71)	0.78 (0.70–0.85)	0.76 (0.72–0.80)	2.7 (2.2–3.5)	0.44 (0.38–0.52)	7 (4–9)
**ACC/AHA**	Overall	0.74 (0.68–0.80)	0.79 (0.43–0.95)	0.88 (0.85–0.91)	22.5 (1.7–300)	0.22 (0.13–0.37)	103 (5–2171)
	Man	0.74 (0.67–0.81)	0.63 (0.50–0.74)	0.75 (0.71–0.79)	1.9 (1.6–2.2)	0.39 (0.34–0.44)	5 (4–6)
	Woman	0.65 (0.59–0.71)	0.70 (0.67–0.72)	0.75 (0.71–0.78)	2.3 (2.1–2.5)	0.44 (0.38–0.51)	5 (4–7)
**SCORE**	Overall	0.77 (0.57–0.89)	0.69 (0.43–0.87)	0.81 (0.77–0.84)	2.3 (1.5–3.6)	0.30 (0.21–0.43)	8 (5–11)
	Man	0.65 (0.47–0.79)	0.95 (0.56–1)	0.78 (0.74–0.82)	8 (1.3–47.8)	0.38 (0.25–0.59)	21 (3–167)
	Woman	0.75 (0.21–0.97)	0.92 (0.74–0.98)	0.91 (0.88–0.93)	7.2 (2.7–19.1)	0.30 (0.09–1)	24 (4–144)
**PCE**	Overall	0.80 (0.66–0.88)	0.68 (0.41–0.86)	NR	NR	NR	NR
	Man	0.85 (0.39–0.98)	0.81 (0.57–0.93)	0.87 (0.84–0.90)	3.9 (1.5–10.2)	0.20 (0.04–1.02)	19 (2–222)
	Woman	0.63 (0.44–0.79)	0.96 (0.47–1.00)	0.77 (0.73–0.81)	10.6 (0.7–162)	0.38 (0.21–0.66)	28 (1–706)
**Cox**	Overall	0.85 (0.81–0.88)	0.80 (0.79–0.81)	0.89 (0.86–0.92)	4.2 (4–4.5)	0.19 (0.15–0.24)	22 (17–31)
	Man	NR	NR	NR	NR	NR	NR
	Woman	NR	NR	NR	NR	NR	NR
**ASCVD**	Overall	NR	NR	NR	NR	NR	NR
	Man	0.60 (0.51–0.69)	0.41 (0.04–0.91)	0.59 (0.55–0.63)	1 (0.4–3)	0.97 (0.21–4.56)	1 (0–14)
	Woman	0.58 (0.41–0.73)	0.80 (0.69–0.88)	0.77 (0.73–0.80)	2.9 (1.6–5.2)	0.53 (0.35–0.81)	5 (2–14)
**WHO**	Overall	NR	NR	NR	NR	NR	NR
	Man	0.23 (0.10–0.44)	0.89 (0.55–0.98)	0.48 (0.43–0.52)	2.4 (0.8–7)	0.82 (0.77–0.88)	3 (1–9)
	Woman	NR	NR	NR	NR	NR	NR
**ATP III**	Overall	0.65 (0.59–0.71)	0.66 (0.64–0.67)	NR	NR	NR	NR
	Man	0.14 (0.14–0.14)	0.86 (0.86–0.86)	NR	NR	NR	NR
	Woman	0.11 (0.11–0.11)	0.89 (0.89–0.89)	NR	NR	NR	NR
**ESC**	Overall	0.79 (0.73–0.84)	0.98 (0.97–0.99)	NR	NR	NR	NR
	Man	0.16 (0.14–0.18)	0.92 (0.91‐‐0.92)	NR	NR	NR	NR
	Woman	0.08 (0.07–0.10)	0.95 (0.95–0.96)	NR	NR	NR	NR
**ASSIGN**	Overall	NR	NR	NR	NR	NR	NR
	Man	0.47 (0.47–0.49)	0.52 (0.51–0.53)	NR	NR	NR	NR
	Woman	0.47 (0.47–0.49)	0.52 (0.52–0.52)	NR	NR	NR	NR
**Reynolds**	Overall	NR	NR	NR	NR	NR	NR
	Man	0.72 (0.68–0.76)	0.67 (0.66–0.69)	NR	NR	NR	NR
	Woman	0.72 (0.67–0.76)	0.68 (0.66–0.69)	NR	NR	NR	NR
**CVD risk score**	Overall	0.72 (0.66–0.77)	0.68 (0.67–0.69)	NR	NR	NR	NR

*Note:* Summary estimates of sensitivity, specificity, area under the receiver operating characteristic curve (AUC), positive likelihood ratio (PLR), negative likelihood ratio (NLR), and diagnostic odds ratio (DOR)

### 3.6 Threshold Effects

Spearman's correlation testing revealed no statistically significant threshold effects for most models: FRS: *ρ* = –0.42, *p* = 0.18; ACC/AHA: *ρ* = –0.35, *p* = 0.29; COX models: *ρ* = –0.28, *p* = 0.41. A borderline result was observed for SCORE (*ρ* = –0.51, *p* = 0.12), suggesting moderate influence from varying cut‐offs (e.g., SCORE‐low 5% vs. SCORE‐high 10%). These findings indicate that threshold heterogeneity did not materially drive the pooled sensitivity and specificity estimates for the majority of models. Sensitivity analyses restricted to the 10% cut‐off (where ≥ 5 studies were available for FRS and SCORE) yielded similar results (e.g., FRS sensitivity 0.78 (95% CI 0.76–0.88) vs. original 0.79), supporting the robustness of the primary estimates. Detailed threshold‐effect results and sensitivity analyses are provided in Supporting File, Table S5.

### 3.7 FRS Model

**Figure 2:**
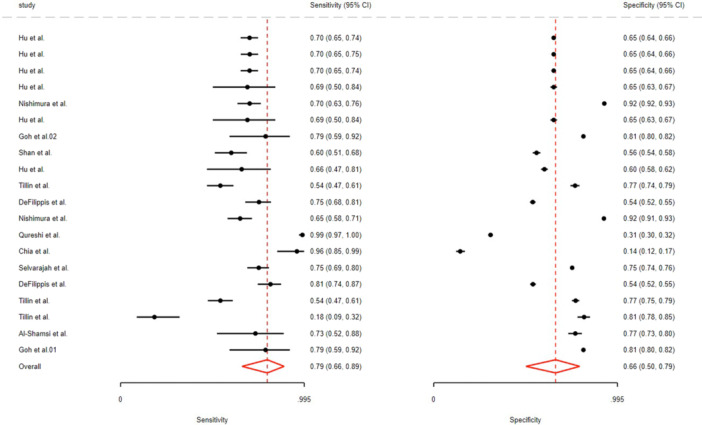
The overall sensitivity and specificity for the FRS model.

Pooled sensitivity and specificity were 0.79 (95% CI: 0.77–0.89) and 0.66 (95% CI: 0.50–0.79), respectively. The overall AUC was 0.77 (95% CI: 0.73–0.80), with values of 0.74 (95% CI: 0.70–0.78) in men and 0.76 (95% CI: 0.72–0.80) in women (Figure 2, Table 2). The SROC curves for the FRS model are presented in the Supporting File, Figure S2a. Pooled PLR was 2.3 (95% CI: 1.8–2.9), NLR 0.40 (95% CI: 0.30–0.52), and DOR 6 (95% CI: 4–9) (Table 2). No publication bias was evident (Deeks’ test p = 0.53; Egger's *p* = 0.53) (Supporting File, Figure S2b). Fagan plot analysis showed post‐test CVD probability rising from 20% to 37% with a positive FRS and falling to 9% with a negative result (Supporting File, Figure S2c). The heterogeneity of the studies was assessed using bivariate boxplots, and the diagnostic accuracy of the index test was evaluated using a scatter matrix, as shown in Supporting File, Figure S2d. and Figure S2e.

### 3.8 ACC/AHA Model

**Figure 3:**
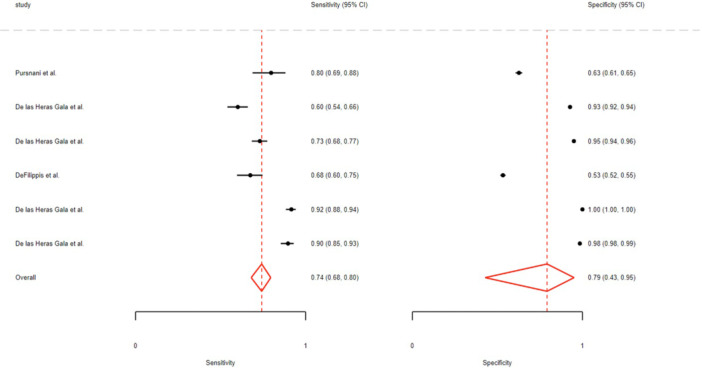
The overall sensitivity and specificity for the ACC/AHA model.

Pooled sensitivity and specificity were 0.74 (95% CI: 0.68–0.80) and 0.79 (95% CI: 0.43–0.95). Sex‐stratified AUCs were 0.87 (95% CI: 0.84–0.90) in men and 0.77 (95% CI: 0.73–0.81) in women (Figure 3 and Table 2). The SROC curves for the ACC/AHA model are presented in the Supporting File, Figure S3a. No publication bias was detected (Deeks’ test *p* = 0.29) (Supporting File, Figure S3b). The Fagan plot showed post‐test probability rising from 20% to 85% (positive) and falling to 5% (negative) (Supporting File, Figure S3c). The heterogeneity of the studies was assessed using bivariate boxplots, and the diagnostic accuracy of the index test was evaluated using a scatter matrix, as shown in Supporting File, Figure S3d and Figure S3e.

### 3.9 SCORE Model

**Figure 4:**
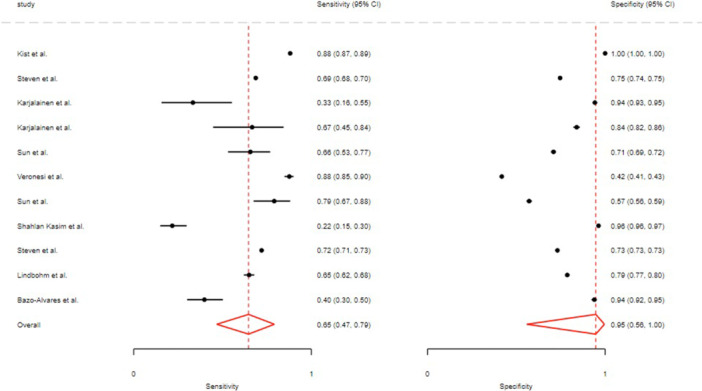
The overall sensitivity and specificity for the SCORE model.

Pooled sensitivity and specificity were 0.77 (95% CI: 0.57–0.89) and 0.69 (95% CI: 0.43–0.89). Overall AUC was 0.81 (95% CI: 0.77–0.84), with values of 0.78 (95% CI: 0.74–0.82) in men and 0.91 (95% CI: 0.88–0.93) in women (Figure 4, Table 2). PLR 2.3 (95% CI: 1.5–3.6), NLR 0.30 (95% CI: 0.21–0.43), DOR 8 (95% CI: 5–11) (Table 2). The SROC curves for the SCORE model are presented in the Supporting File, Figure S4a. No publication bias (Deeks' test *p* = 0.88) (Supporting File, Figure S4b). The Fagan plot showed post‐test probability rising from 20% to 36% (positive) and falling to 7% (negative) (Supporting File, Figure S4c). The consistency across studies was examined using bivariate boxplots, and the diagnostic precision of the index test was analyzed through a scatter matrix, as illustrated in Supporting File, Figure S4d and Figure S4e.

### 3.10 Pooled‐ Cohort Equation (PCE) Model

The overall sensitivity and specificity of the PCE model were determined to be 0.80 (95% CI: 0.66–0.88) and 0.68 (95% CI: 0.41–0.86), respectively. The AUC for the overall SROC curve of the PCE model was not provided. However, the AUC values were reported as 0.87 (95% CI: 0.84–0.90) for men and 0.77 (95% CI: 0.73–0.81) for women. The pooled predictive metrics for the PCE model included a PLR, NLR, and DOR, which were 3.9, 0.20, and 19, respectively, for men, and 10.6, 0.38, and 28, respectively, for women, as detailed in Table 2.

### 3.11 COX Model

Pooled sensitivity and specificity were 0.85 (95% CI: 0.81–0.88) and 0.80 (95% CI: 0.79–0.81). Overall AUC was 0.89 (95% CI: 0.89–0.92), the highest among major models. PLR, NLR, and DOR are detailed in Table 2.

### 3.12 ASCVD Model

The diagnostic meta‐analysis for the ASCVD model in predicting CVD indicated a sensitivity of 0.60 (95% CI: 0.51–0.69) for men and 0.58 (95% CI: 0.41–‐0.73) for women, with specificity values of 0.41 (95% CI: 0.04–0.91) for men and 0.80 (95% CI: 0.69–0.88) for women. The AUC for the overall SROC curve of the ASCVD model was not provided. However, the AUC was reported as 0.59 (95% CI: 0.55–0.63) for men and 0.77 (95% CI: 0.73–0.80) for women, as detailed in Table 2.

### 3.13 WHO Model

The WHO model has been documented exclusively for studies involving men. The diagnostic meta‐analysis for this model in predicting CVD reported a sensitivity of 0.23 (95% CI: 0.10–0.44) and a specificity of 0.89 (95% CI: 0.55–0.94). The AUC for the WHO model was 0.48 (95% CI: 0.43–0.52) for men (Table 2).

### 3.14 Other Models

Results for ATP III, ESC, ASSIGN, Reynolds, and CVD Risk Score are summarized in Table 2. Reynolds showed consistent sensitivity (~0.72) across groups, while ESC exhibited lower sensitivity but higher specificity (Table 2).

### 3.15 Subgroup Analysis and Meta‐Regression

**Figure 5:**
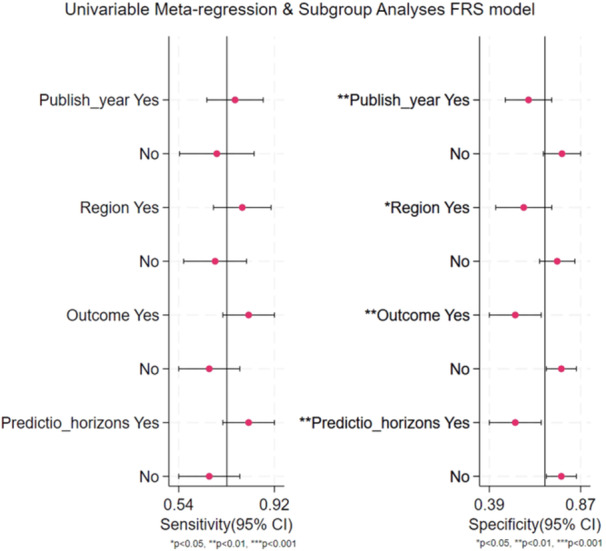
The source of heterogeneity. Univariable meta‐regression analysis.

The heterogeneity of the studies was assessed using bivariate boxplots in the FRS model. As shown in Supporting File, Figure S5, six studies were not included in the boxplots. This suggests that these six studies may be the underlying cause of heterogeneity. After carefully reading these six studies, the key influencing factors of heterogeneity were identified: region, outcome, prediction horizon, and year of publication. Subsequently, we isolated these six studies from all studies to create separate subgroups for analysis. As shown in Figure 5, region, outcome, prediction horizons, and year of publication were sources of heterogeneity, either in sensitivity or specificity (*p* < 0.05) (Figure 5). We did not perform meta‐regression analysis to explore potential heterogeneity factors for other models due to the limitation of the number of validation studies. Generally, at least 10 reports were needed for meta‐regression to obtain valid results.

## 4 Discussion

To the best of our knowledge, this is the first meta‐analysis specifically designed to evaluate the diagnostic accuracy of CVD prediction models. The study reviewed a total of 58 cohort studies, involving 7.5 million participants, with recorded CVD events numbering 47,276 among men and 33,931 among women. Findings revealed that the overall sensitivity and specificity were 0.79 and 0.66 for the FRS model, 0.74 and 0.79 for the ACC/AHA model, 0.77 and 0.69 for the SCORE model, and 0.85 and 0.80 for the COX model, respectively. The overall area under the curve (AUC), which combines sensitivity and specificity, reached 0.91 for the SCORE model in men and 0.87 for the PCE model in women.

In comparison with other studies assessing optimal models for CVD prediction across diverse populations, the study by Sajeev et al. [14]. evaluated models such as Framingham, Globorisk, and QRISK3 for CVD prediction. Their results indicated that QRISK3, owing to its comprehensive risk factor inclusion and utilization of larger datasets, demonstrated greater potential for accuracy and sensitivity in diverse populations. Other models also performed acceptably, depending on local data and sample size.

The study by Goh et al. [68], which compared the Framingham and SCORE models (for low‐ and high‐risk European regions) in predicting CVD mortality among 4487 Australian women, reported a sensitivity of approximately 67% for FRS, with AUC values of 0.85, 0.67, and 0.87 for the FRS, SCORE‐Low, and SCORE‐High models, respectively. The SCORE‐High model exhibited the highest AUC, but Framingham was recommended due to its better calibration and broader applicability for 10‐year CVD mortality prediction in Australian women. The SCORE model, a well‐established tool, has been evaluated in various populations, with an AUC of 0.77 reported in Malaysia and 0.76 and 0.78 among Austrian men and women, respectively.

In a study by Birhanu et al. [81] which compared the World Health Organization Risk Score (WHO‐RS), Australian Risk Score (ARS), and Globorisk tools among 40‐ to 74‐year‐old participants without prior CVD in a prospective cohort study in Indian villages, the ARS showed a sensitivity of 48% and specificity of 75%–80%, while the WHO‐RS had a sensitivity of 35%–40% and specificity of 75%–80%. For the Globorisk Lab and Non‐Lab models, sensitivity ranged from 35% to 40%, with a specificity of 75%–80%. The WHO‐RS tended to underestimate cardiovascular risk, whereas the ARS overestimated it, particularly among men. Both Globorisk variants demonstrated relatively good discrimination and agreement.

A meta‐analysis by Liu et al. [82] comparing machine learning (ML) models with traditional CVD risk prediction models found that Random Forest and Deep Learning outperformed others, with combined AUC of 0.88 (95% CI: 0.81–0.91) and 0.84 (95% CI: 0.77–0.92), respectively. Ensemble methods like Gradient Boosting Machine (GBM) also performed well, with an AUC of 0.821 (95% CI: 0.77–0.86). Traditional models such as QRISK3 and ASCVD showed lower performance, with combined AUCs of 0.77 (95% CI: 0.69–0.85) and 0.75 (95% CI: 0.66–0.84), respectively. Logistic regression and Cox models had AUCs of 0.796 (95% CI: 0.730–0.842) and 0.787 (95% CI: 0.740–0.834), respectively, while the PCE model had an AUC of 0.765 (95% CI: 0.71–0.79). Overall, ML models, particularly Random Forest and Deep Learning, offered higher accuracy, sensitivity, and AUC due to their ability to analyze extensive electronic health record (EHR) data and adapt to patient diversity, though limitations such as heterogeneity and the need for further validation persist.

In a study by Bazo‐Alvarez et al. [80] comparing CVD risk prediction models across five Peruvian regions, the ACC/AHA model exhibited the highest sensitivity and specificity at 0.71 each, outperforming FRS, SCORE, WHO Risk Chart, and Sex and Hypertension Risk (SHR) models, which averaged around 0.65. Lim et al.'s study, comparing FRS and ASCVD performance in predicting CVD risk among South Korean populations, reported sensitivities of 63.8% and 73% for FRS in men and women, respectively, and 61.2% and 61.7% for ASCVD. Specificities were 75.7% and 64.6% for FRS, and 71.6% and 72.8% for ASCVD in men and women, respectively. FRS outperformed ASCVD, with AUCs of 0.75 and 0.74 for FRS in men and women, compared to 0.71 and 0.72 for ASCVD.

Zhang et al. [83] investigated CVD risk assessment tools, focusing on demographic differences between Western and Asian populations, finding that Western models like Framingham, SCORE, PCE, and QRISK3 are unsuitable for Asian populations without recalibration due to differing risk factor profiles (e.g., hypertension and diabetes). Models such as China‐PAR and JALS, tailored to local profiles, were recommended for Asia, as Western models were developed based on large European and American cohorts.

A meta‐analysis by Nomali et al. [84] on 10‐year CVD risk prediction models in Asian populations found that Framingham CHD, general Framingham CVD, PCE, SCORE, Globorisk, and WHO risk charts were externally validated in East Asia. The general Framingham CVD and PCE models were the most reliable, while Globorisk and WHO were validated once. All models showed acceptable discrimination in both genders, though the Framingham CHD model overestimated risk in both, the general Framingham CVD and SCORE overestimated in men and underestimated in women, and PCE underestimated in both.

A systematic review and meta‐analysis by Damen et al. [85] evaluated the Framingham CHD and PCE models, while in 2022, Zhiting et al. assessed the general Framingham CVD and PCE models in the Chinese general population. Both reviews focused on the most reliable models [8]. A CVD risk model can estimate the likelihood of specific cardiac events based on the presence or absence of cardiovascular risk factors, with discrimination and calibration being the most widely used statistical evaluation methods, as confirmed in other studies [17].

The prediction models reviewed, including Framingham, SCORE, QRISK2, and others, were developed specifically for predicting CVD or CHD in the general population, typically incorporating known risk factors such as age, sex, blood pressure, cholesterol, and smoking history. However, variations in International Classification of Diseases (ICD‐9 or ICD‐10) codes for identifying CVD or CHD events across studies may affect model accuracy [12].

The FRS performs well in terms of discrimination and calibration among White and Black populations in the United States, but only shows acceptable discrimination in Asian‐American, Native American, Hispanic‐American, and Native Chinese populations, often overestimating cardiovascular risk. In non‐White populations, FRS may overestimate risk by 50%, limiting its applicability in many non‐Western settings. In some low‐risk European regions, FRS may also overestimate risk [2, 82, 86].

Most models are calibrated for specific populations, such as adults over 40 or those with moderate‐to‐high risk, potentially reducing their accuracy in younger or low‐risk groups. Models using data from larger cohorts, like Framingham or QRISK2, tend to have higher accuracy but may lack sufficient geographic or ethnic diversity, necessitating further evaluation for generalizability across populations [87, 88].

Identifying new risk factors is a critical aspect of cardiovascular research. Assessing the incremental value of each prediction model may reveal additional insights. Incorporating new, significant variables into CVD risk models could enhance knowledge, leading to optimized management of patients with moderate risk. Furthermore, evaluating model performance using populations not involved in risk score derivation, including new cohorts, offers valuable insights.

Other studies have highlighted the existence of various CVD prediction models in the general population, a finding consistent with prior reviews. However, most risk prediction models were developed in predominantly White European populations, limiting their utility for other ethnic groups. Many reviews conclude that while numerous prediction models exist, there is a lack of consensus on the most appropriate model for decision‐making.

The high heterogeneity observed in our pooled estimates (I² > 50% for most performance metrics) is consistent with previous meta‐analyses of CVD risk prediction models, reflecting the diversity in study populations, model adaptations, and methodological approaches. Meta‐regression analysis revealed that sample size and publication year were the main sources of heterogeneity, with smaller studies and older publications contributing to greater variability in sensitivity and specificity (*p* < 0.05). This aligns with findings from Guo et al.‘s systematic review of CVD risk models in the Chinese population, where similar heterogeneity was attributed to differences in cohort sizes and temporal changes in risk factor profiles, leading to varying discrimination (AUC ranging from 0.60 to 0.83) [82]. Our subgroup analyses, stratifying by region, outcome definition, prediction horizon, and publication year, confirmed significant effects on sensitivity for non‐Western populations and composite outcomes, underscoring the need for localized recalibration. These sources of heterogeneity highlight limitations in generalizing Western‐derived models like FRS to diverse ethnic groups, as seen in the China‐PAR model's superior calibration in Chinese cohorts [89]. Future studies should prioritize larger, multi‐ethnic validations and standardized reporting to reduce heterogeneity and enhance model applicability.

We recommend recalibrating CVD prediction models using local and regional data to improve their accuracy in specific populations. Advanced models, such as those employing machine learning, may offer superior performance due to their ability to analyze complex data, though they require extensive validation. Transparent reporting of methods and results in future studies is essential to facilitate comparison and continuous improvement of these models.

Threshold heterogeneity did not materially affect pooled estimates for most models based on non‐significant Spearman correlations, but the borderline result for SCORE highlights this as a core limitation, potentially contributing to observed heterogeneity and underscoring the need for standardized cut‐offs in future validations.

Strengths of this study include the evaluation of various CVD prediction models across diverse populations and the estimation of pooled sensitivity, specificity, and AUC for each model overall and by gender. Other studies have used discrimination and calibration for statistical evaluation of CVD prediction models. Limitations include the limited number of studies available for certain models, which necessitated combining similar models to enable the calculation of pooled sensitivity, specificity, and AUC.

## 5 Conclusions

This systematic review and meta‐analysis of diagnostic accuracy assessed various models for predicting cardiovascular disease. The findings indicate that the FRS, SCORE, and COX‐based models demonstrated superior sensitivity and specificity. Based on pooled AUCs, the most reliable threshold‐independent metric, SCORE, and COX demonstrated superior performance, followed by FRS. While sensitivity/specificity and DOR provide classification insights, AUC offers the most robust basis for cross‐model comparison. However, the clinical utility of most models remains uncertain owing to methodological limitations, incomplete reporting, and a lack of external validation and impact studies. In an era of abundant large‐scale datasets, rather than creating additional similar risk prediction models, future research should prioritize external validation and direct head‐to‐head comparisons of existing promising models, their adaptation or combination to local populations, and investigation of whether incorporating novel predictors can enhance their performance.

## Author Contributions

**Mohammad Aziz Rasouli:** investigation, writing – original draft, writing – review and editing, validation, methodology, formal analysis, data curation. **Farid Najafi:** conceptualization, writing – review and editing, methodology, data curation. **Alireza Ansari‐Moghaddam:** conceptualization, writing – review and editing, data curation, visualization. **Bijan Nouri:** conceptualization, methodology, writing – review and editing, data curation. **Farhad Moradpour:** conceptualization, writing – review and editing, data curation. **Ghobad Moradi:** conceptualization, writing – original draft, writing – review and editing, methodology, supervision, data curation, validation. **Yousef Moradi:** conceptualization, investigation, writing – original draft, writing – review and editing, methodology, validation, data curation, supervision.

## Funding

The authors have nothing to report.

## Ethics Statement

The authors have nothing to report.

## Consent

The authors have nothing to report.

## Conflicts of Interest

The authors declare no conflicts of interest.

## Transparency Statement

The lead authors, Ghobad Moradi and Yousef Moradi, affirm that this manuscript is an honest, accurate, and transparent account of the study being reported; that no important aspects of the study have been omitted; and that any discrepancies from the study as planned (and, if relevant, registered) have been explained.

## Supporting information

Supporting File

## Data Availability

The original contributions presented in the study are included in the article/Supporting Material. Further inquiries can be directed to the corresponding author.
