# Supplementary material for: Diagnostic Accuracy of Cardiovascular Disease Prediction Models: A Systematic Review and Meta‐Analysis of Validation Studies
Source: Health Sci Rep. 2026 Apr 29;9(5):e72469. doi: 10.1002/hsr2.72469 (PMC13129226; doi:10.1002/hsr2.72469)

**Supplementary File**

**Table S1.** Search Strategy Overview

| Category | Details |
| --- | --- |
| Search Terms | “cardiovascular disease” , “ CVD ”, “cardiovascular ” , “heart disease” , “extremely drug resistant” , “cardiac disease” , “carbapenem-resistant” , “stroke”, “clinical” , “coronary heart disease” , “CHD” , “risk predic” , “ risk scor” , “risk calculation” , “ risk assessment ” |
| Databases Searched | Medline (via PubMed), Scopus, Web of Science, Cochrane Library, EMBASE, and CINAHL, |
| Search Terms and Boolean Logic | |
| PubMed (Medline) | (("cardiovascular disease*"[Title] OR "CVD"[Title] OR "cardiovascular*"[Title] OR "heart disease*"[Title] OR "cardiac disease*"[Title] OR "cardiac disorder*"[Title] OR "coronary disease"[Title] OR "coronary heart disease*"[Title] OR "CHD"[Title] OR "cerebrovascular*"[Title] OR "stroke"[Title]) AND ("risk predic*"[Title] OR "risk scor*"[Title] OR "risk calculation"[Title] OR "risk assessment"[Title])) AND (2013:2025[pdat]) |
| EMBASE | (('cardiovascular disease*':ti OR 'CVD':ti OR 'cardiovascular*':ti OR 'heart disease*':ti OR 'cardiac disease*':ti OR 'cardiac disorder*':ti OR 'coronary disease':ti OR 'coronary heart disease*':ti OR 'CHD':ti OR 'cerebrovascular*':ti OR 'stroke':ti) AND ('risk predic*':ti OR 'risk scor*':ti OR 'risk calculation':ti OR 'risk assessment':ti)) AND ([2013-2025]/py) |
| Scopus | (TITLE("cardiovascular disease*" OR "CVD" OR "cardiovascular*" OR "heart disease*" OR "cardiac disease*" OR "cardiac disorder*" OR "coronary disease" OR "coronary heart disease*" OR "CHD" OR "cerebrovascular*" OR "stroke") AND TITLE("risk predic*" OR "risk scor*" OR "risk calculation" OR "risk assessment")) AND PUBYEAR > 2012 AND PUBYEAR < 2025 |
| Web of Science | TI=("cardiovascular disease*" OR "CVD" OR "cardiovascular*" OR "heart disease*" OR "cardiac disease*" OR "cardiac disorder*" OR "coronary disease" OR "coronary heart disease*" OR "CHD" OR "cerebrovascular*" OR "stroke") AND TI=("risk predic*" OR "risk scor*" OR "risk calculation" OR "risk assessment") AND PY=(2013-2025) |
| Cochrane Library | ("cardiovascular disease*":ti OR "CVD":ti OR "cardiovascular*":ti OR "heart disease*":ti OR "cardiac disease*":ti OR "cardiac disorder*":ti OR "coronary disease":ti OR "coronary heart disease*":ti OR "CHD":ti OR "cerebrovascular*":ti OR "stroke":ti) AND ("risk predic*":ti OR "risk scor*":ti OR "risk calculation":ti OR "risk assessment":ti) in Title with Publication Year from 2013 to 2025 |
| CINAHL | TI ("cardiovascular disease*" OR "CVD" OR "cardiovascular*" OR "heart disease*" OR "cardiac disease*" OR "cardiac disorder*" OR "coronary disease" OR "coronary heart disease*" OR "CHD" OR "cerebrovascular*" OR "stroke") AND TI ("risk predic*" OR "risk scor*" OR "risk calculation" OR "risk assessment") AND PY 2013-2025 |
| Grey Literature Search Results | ("cardiovascular disease*" OR "CVD" OR "cardiovascular*" OR "heart disease*" OR "cardiac disease*" OR "cardiac disorder*" OR "coronary disease" OR "coronary heart disease*" OR "CHD" OR "cerebrovascular*" OR "stroke") ("risk predic*" OR "risk scor*" OR "risk calculation" OR "risk assessment") |
| Search Deadline | From January 1, 2013, to December 30, 2024 |

**Table S2.** Study Eligibility Criteria PIRT (Population, Index test, Reference test, Target condition)

| Category | Inclusion Criteria | Exclusion Criteria |
| --- | --- | --- |
| Population | Individuals with known risk factors for cardiovascular disease, individuals with a history of cardiovascular events, or the general population. | Studies focusing exclusively on specific subgroups with pre-existing conditions (e.g., individuals with diabetes, HIV, atrial fibrillation, or those undergoing cardiac procedures), rather than the general population or broad age/region cohorts aimed at primary CVD prevention.  Research lacking a clearly defined study population or involving populations not representative of all ages and regions targeted for primary prevention. |
| Index Test | This is the test or model whose accuracy is being evaluated. For CVD prediction, this could be a specific risk score (e.g., Framingham Risk Score, QRISK), a new biomarker, or a combination of clinical and demographic factors. | Systematic reviews, reviews, case series, and case reports evaluating CVD prediction models.  Studies with incomplete data where sensitivity, specificity, or optimal diagnostic thresholds cannot be calculated or reported.  Research assessing the added value of new predictors to established models, studies examining a single predictor, or models developed/validated exclusively in specific disease subgroups (e.g., diabetes, HIV).  Challenged or withdrawn literature related to the index test (CVD prediction models like FRS, SCORE, etc.). |
| Reference Test | This is the established standard used to confirm the presence or absence of the target condition (CVD). It serves as the "gold standard" for determining whether the index test correctly identifies individuals with CVD or those at risk. | Studies lacking a clearly defined endpoint or diagnostic criteria for CVD, such as those focusing solely on models predicting the risk of venous disease or stroke alone, without a composite CVD outcome.  Research where the reference standard (e.g., clinical diagnosis of CVD events) is not adequately described or validated. |
| Target Condition | This is the specific CVD outcome of interest. For CVD prediction models, this might be the occurrence of a major adverse cardiovascular event (MACE) (e.g., heart attack, stroke, cardiovascular death), a specific type of CVD (e.g., coronary artery disease, heart failure), or a certain level of risk for these events. | Studies not aligned with the primary prevention of CVD, including those addressing secondary prevention or unrelated conditions.  Investigations targeting conditions outside the scope of general CVD (e.g., venous thromboembolism or isolated stroke risk) rather than a broad CVD outcome (e.g., myocardial infarction, stroke, CVD death). |

**Table S3.** Quality assessment of included studies based on the revised tool for the quality assessment of diagnostic accuracy studies checklist

| **Study** | **Risk of Bias** | | | |  | **Applicability Concerns** | | |
| --- | --- | --- | --- | --- | --- | --- | --- | --- |
|  | **PATIENT SELECTION** | **INDEX TEST** | **REFERENCE STANDARD** | **FLOW AND TIMING** |  | **PATIENT SELECTION** | **INDEX TEST** | **REFERENCE STANDARD** |
| Kist (2023) | Low | Low | Low | Low |  | Low | Low | Low |
| Angelow (2022) | Unclear | Low | High | Unclear |  | Low | Low | Low |
| Steven (2023) | Unclear | Low | Unclear | Low |  | Low | Low | Low |
| Pylypchuk (2018) | Low | Low | Unclear | Low |  | Low | Low | Low |
| Backholer (2017) | Unclear | Low | Low | Low |  | Low | Low | Low |
| Artigao-Rodenas (2013) | Unclear | Low | Low | Unclear |  | Low | Low | Low |
| Al-Shamsi (2020) | Unclear | Low | Unclear | Unclear |  | High | Low | Low |
| Shan (2022) | Unclear | Unclear | Unclear | Unclear |  | High | Low | Unclear |
| Qiu (2023) | Low | Low | Unclear | High |  | High | Low | Low |
| Lindbohm (2021) | High | Unclear | Unclear | High |  | High | Low | Low |
| Lim (2024) | Low | Unclear | Low | Low |  | Low | Low | Low |
| Stenling (2020) | Low | Unclear | Low | Low |  | Low | Low | Low |
| Hsu (2024) | Unclear | Unclear | Low | Low |  | Low | Low | Low |
| Chun (2018) | Low | Unclear | Low | Low |  | Low | Low | Low |
| Shahlan Kasim (2023) | Low | Unclear | Low | Low |  | Low | Low | Low |
| Husein (2019) | Low | Low | Unclear | Low |  | Low | Low | Low |
| Mansoor (2019) | Low | Unclear | Low | Low |  | Low | Low | Low |
| Yang (2016) | Low | Unclear | Low | Low |  | Low | Low | Low |
| Veronesi (2017) | Unclear | Unclear | Low | Low |  | Low | Low | Low |
| Van Staa (2014) | Low | Unclear | Low | Low |  | Low | Low | Low |
| Tralhao (2016) | High | Unclear | Low | Low |  | High | Low | Low |
| Tillin (2014) | Low | Unclear | Low | Low |  | Low | Low | Low |
| Sussman (2017) | Low | Unclear | Low | Low |  | High | Low | Low |
| Sun (2017) | Unclear | Unclear | Unclear | Low |  | High | Low | Low |
| Selvarajah (2014) | Unclear | Unclear | Unclear | Unclear |  | High | Low | Unclear |
| Sarrafzadegan (2017) | Low | Low | Unclear | Low |  | Low | Low | Low |
| Qureshi (2016) | Low | Low | Unclear | Low |  | Low | Low | Low |
| Pursnani (2015) | Unclear | Low | Low | Unclear |  | Low | Low | Low |
| Pylypchuk (2018) | Low | Low | Low | Unclear |  | Low | Low | Low |
| Nishimura (2014) | Unclear | Low | Unclear | Unclear |  | Low | Low | Low |
| Mortensen 1 (2017) | Unclear | Low | Unclear | Unclear |  | Low | Low | Low |
| Mortensen 2 (2015) | Unclear | Low | Unclear | Unclear |  | Low | Low | Low |
| Marrugat (2014) | Unclear | Low | Unclear | Unclear |  | Low | Low | Low |
| Lee (2015) | Unclear | Unclear | Unclear | Unclear |  | Low | Low | Low |
| Lee (2014) | Low | Low | Unclear | Low |  | Low | Low | Low |
| Kempf (2016) | Unclear | Low | Unclear | Low |  | High | Low | High |
| Kavousi (2014) | Low | Low | Low | Unclear |  | High | Low | Low |
| Karjalainen (2017) | Low | Low | Unclear | Low |  | Low | Low | Low |
| Kariuki (2017) | Low | Low | Low | Low |  | Low | Low | Low |
| Jung (2015) | High | Low | Low | Low |  | Low | Low | Low |
| Johansson (2016) | Low | Low | Low | Low |  | Low | Low | Low |
| Jee (2014) | High | Low | Unclear | Low |  | High | Low | Unclear |
| Hua (2017) | High | Low | Low | Low |  | High | Low | Low |
| Hu (2016) | Unclear | Low | Low | Low |  | Low | Low | Low |
| Harari (2017) | Unclear | Low | Unclear | Low |  | High | Low | Low |
| Goh1 (2014) | Low | Low | Low | Low |  | Low | Low | Low |
| Goh 2 (2014) | Low | Low | Unclear | Unclear |  | Low | Low | Low |
| Fox (2016) | Low | Low | Low | Unclear |  | Low | Low | Low |
| Foraker (2016) | Low | Low | Unclear | Low |  | Low | Low | Low |
| Flueckier (2018) | Low | Low | Low | Low |  | Low | Low | Low |
| Fatema (2016) | Low | Low | Low | Unclear |  | Low | Low | Low |
| Dufouil (2017) | Low | Low | Low | Low |  | Low | Low | Low |
| De las Heras Gala (2016) | Low | Low | Low | Low |  | Low | Low | Low |
| DeGoma (2013) | Unclear | Low | Unclear | Unclear |  | Unclear | Low | Unclear |
| DeFilippis (2015) | Low | Low | Low | Low |  | Low | Low | Low |
| Chia (2014) | Unclear | Low | Unclear | Unclear |  | High | Low | Low |
| Boateng (2018) | Unclear | Low | Unclear | Unclear |  | High | Low | Unclear |
| Bazo-Alvares (2015) | Unclear | Low | Unclear | Unclear |  | High | Low | Unclear |


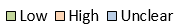


**Figure S1.** Quality assessment of included studies based on the revised tool for the quality assessment of diagnostic accuracy studies checklist


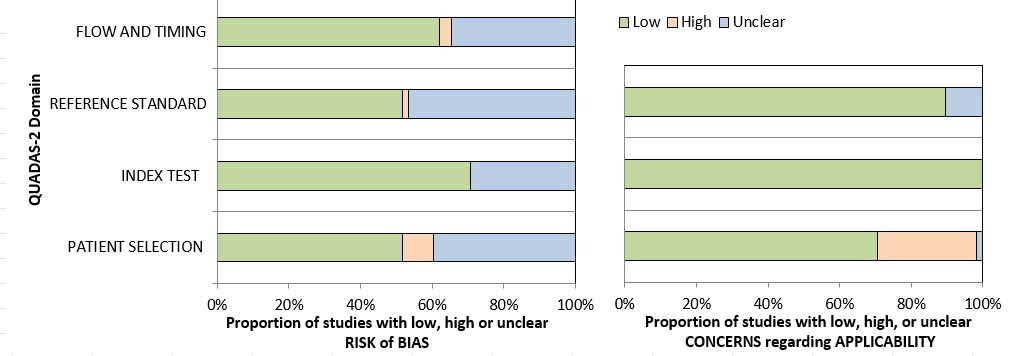


**Table S4**. Critical appraisal of selected prediction modeling studies based on the PROBAST checklist (n =47)

| Overall | | Applicability | | | Risk of bias | | | |  |
| --- | --- | --- | --- | --- | --- | --- | --- | --- | --- |
| Applicability | Risk of bias | Outcome | Predictors | Participant selection | Analysis | Outcome | Predictors | Participant selection | Risk calculator |
| + | + | + | + | + | + | + | + | + | **FRS** |
| + | + | + | + | + | + | + | + | + | **SCORE2** |
| + | + | + | + | - | + | + | + | - | **RPCE** |
| + | + | + | + | - | + | + | + | + | **ASCVD** |
| + | + | + | + | - | + | + | + | - | **Cox** |
| + | + | + | + | + | + | + | + | + | **PCE** |
| + | + | + | ? | ? | + | + | ? | ? | **ATP III** |
| + | + | + | + | ? | + | + | + | ? | **RRS** |
| + | + | + | + | ? | + | + | + | ? | **China- PAR** |
| + | + | + | + | + | + | + | + | + | **WHO** |
| + | + | + | ? | ? | + | + | ? | ? | **ARIC** |
| + | + | + | + | + | + | + | + | + | **CN-ICVD** |
| + | + | + | + | + | + | + | + | + | **CVD risk score** |
| + | + | + | ? | + | + | + | ? | + | **CVDMCM** |
| + | + | + | + | ? | + | + | + | + | **CVH metric** |
| + | + | + | + | + | + | + | + | ? | **EZ-CVD** |
| + | + | + | + | + | + | + | + | + | **Finrisk** |
| + | + | + | ? | + | + | + | ? | + | **Health 2000** |
| + | + | + | + | + | + | + | + | + | **Hybrid** |
| + | + | + | + | ? | + | + | + | + | **KRPM** |
| + | + | + | + | + | + | + | + | + | **ARIC- CHD** |
| + | + | + | ? | + | + | + | ? | ? | **NHNES III- CHD** |
| + | + | + | + | + | + | + | + | + | **O-RSRS** |
| + | + | + | + | + | + | + | + | + | **PARS risk chart** |
| + | + | + | + | ? | + | + | + | + | **Pooled cohort risk score** |
| + | + | + | ? | + | + | + | ? | + | **PRSOCAM** |
| + | + | + | + | + | + | + | + | + | **R-FSRS** |
| + | - | + | + | + | + | + | + | ? | **TC suita** |
| + | - | + | + | + | + | + | + | ? | **Trial-based** |
| + | + | + | ? | + | + | + | ? | ? | **VARS - CVD** |
| + | + | + | + | ? | + | + | + | ? | **Wu’s Simplified Model** |
| + | + | + | + | - | + | + | + | - | **ACC/AHA (PCE)** |
| + | + | + | + | - | + | + | + | - | **American CVH** |
| ? | + | + | + | ? | + | + | ? | + | **ASSIGN** |
| + | + | + | ? | ? | + | + | ? | ? | **ESC/SCORE** |
| + | + | + | + | ? | + | + | + | ? | **FINRISK** |
| + | + | + | + | ? | + | + | + | ? | **Framingham** |
| + | + | + | + | ? | + | + | + | ? | **Health 2000** |
| - | + | + | - | - | + | + | - | - | **Korean Heart study** |
| ? | + | + | - | ? | + | + | - | ? | **Korean Risk Prediction model** |
| + | + | ? | + | + | + | ? | ? | ? | **PARS** |
| + | + | + | + | + | + | + | + | + | **PREDICT** |
| + | + | + | + | ? | + | + | + | ? | **PROCAM** |
| + | + | + | ? | + | + | + | ? | + | **QRISK 3** |
| + | + | + | + | ? | + | + | + | ? | **Reynolds** |
| ? | + | + | + | ? | + | + | ? | ? | **SUITA** |
| + | ? | ? | + | + | + | ? | ? | ? | **VARS CV** |

**Table S5.** Threshold Effects

| Predictive Model | Number of Studies | Spearman's Correlation (ρ) between Sensitivity and (1-Specificity) | P-value for Threshold Effect |
| --- | --- | --- | --- |
| FRS | 18 | –0.42 | 0.18 |
| ACC/AHA | 12 | –0.35 | 0.29 |
| SCORE | 7 | –0.51 | 0.12 |
| COX models | 4 | –0.28 | 0.41 |
| Other models (ASCVD, WHO, ATP III, ESC, etc.) | <5 per model | Not reported | Not reported |

**Figure S2.** FRS (Overall)

| **S1.a.** SROC curve  **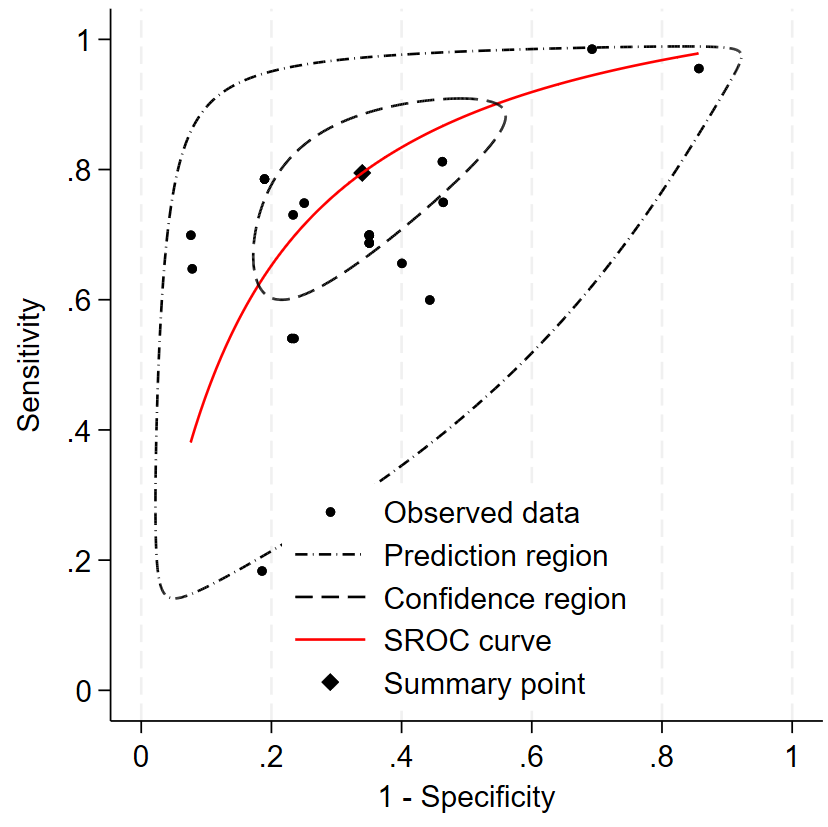** |
| --- |
| **S2.b. Funnel plot**  **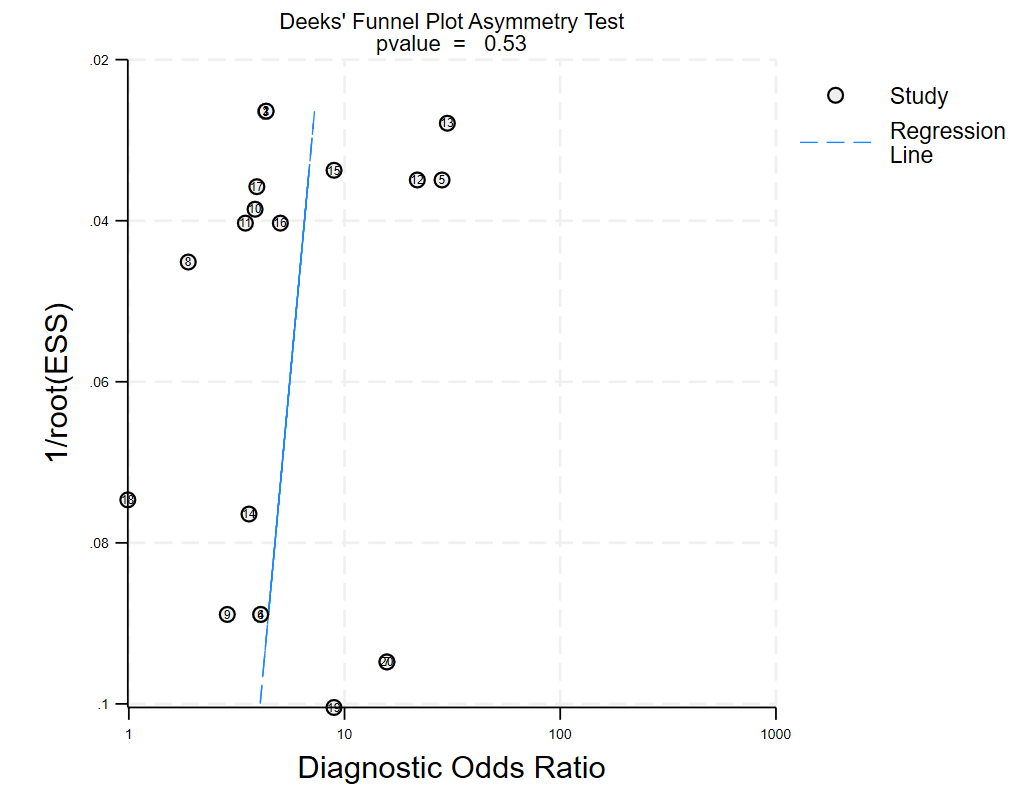** |
| **S1.c. Fagan’s plot**  **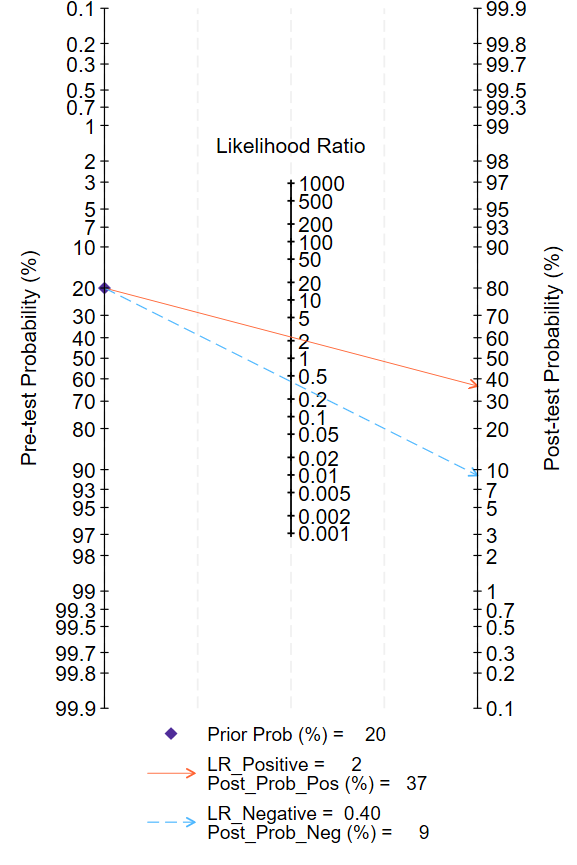** |
| **S1.d. Bivariate boxplot**  **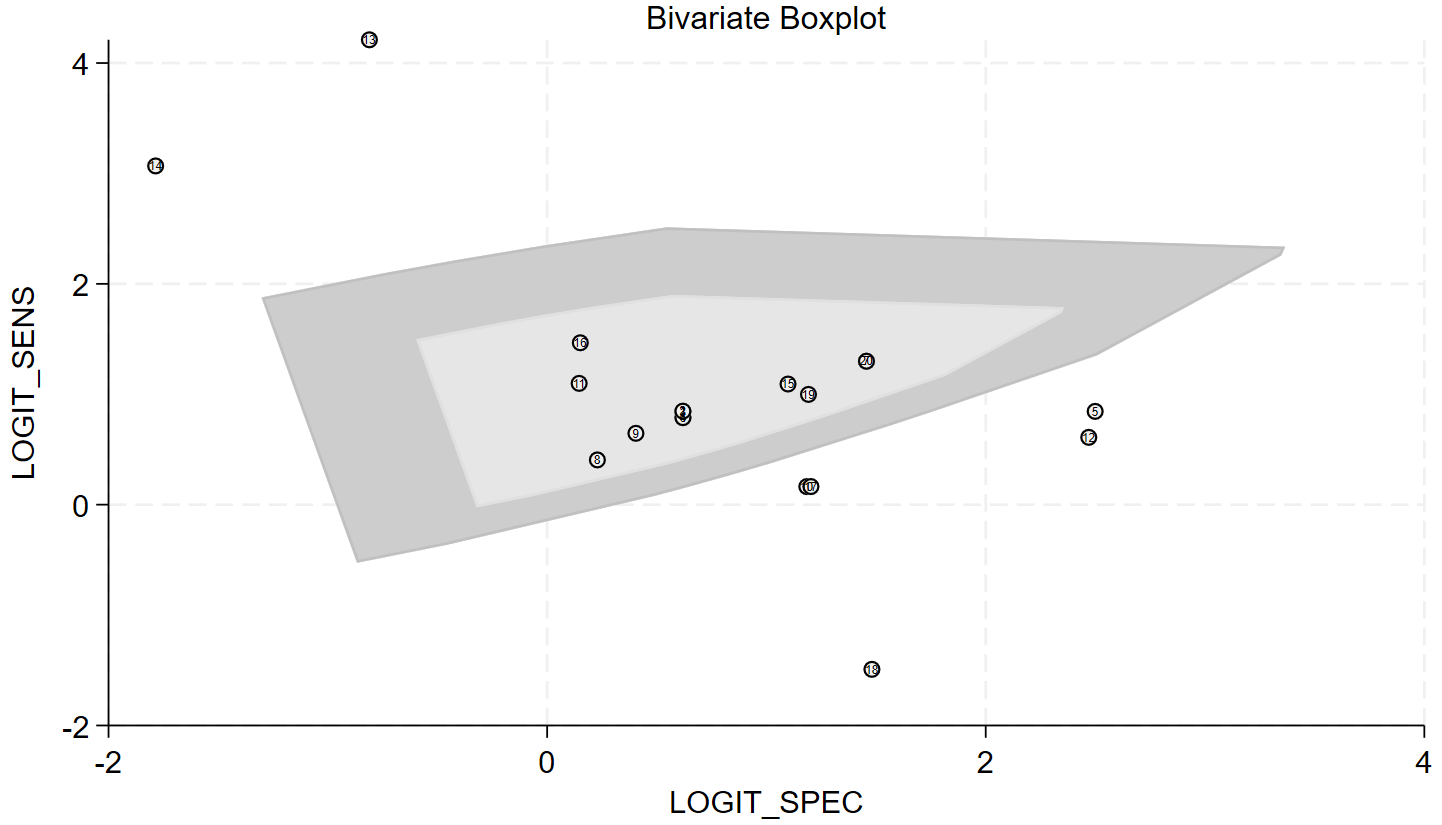** |
| **S2.e.** **Scatter matrix**  **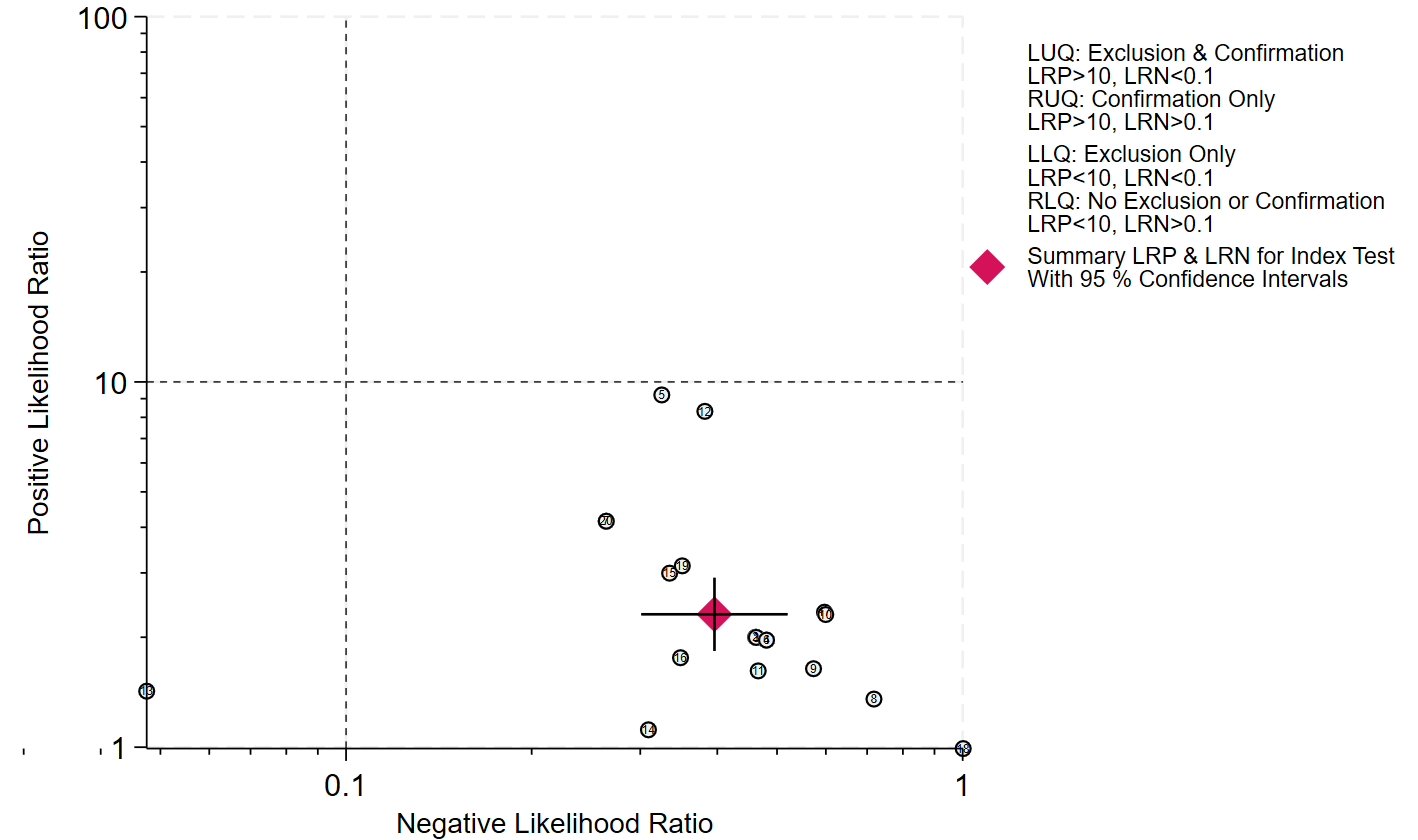** |

**Figure S3.** ACC/AHA model (Overall)

| **S3.a.**  SROC  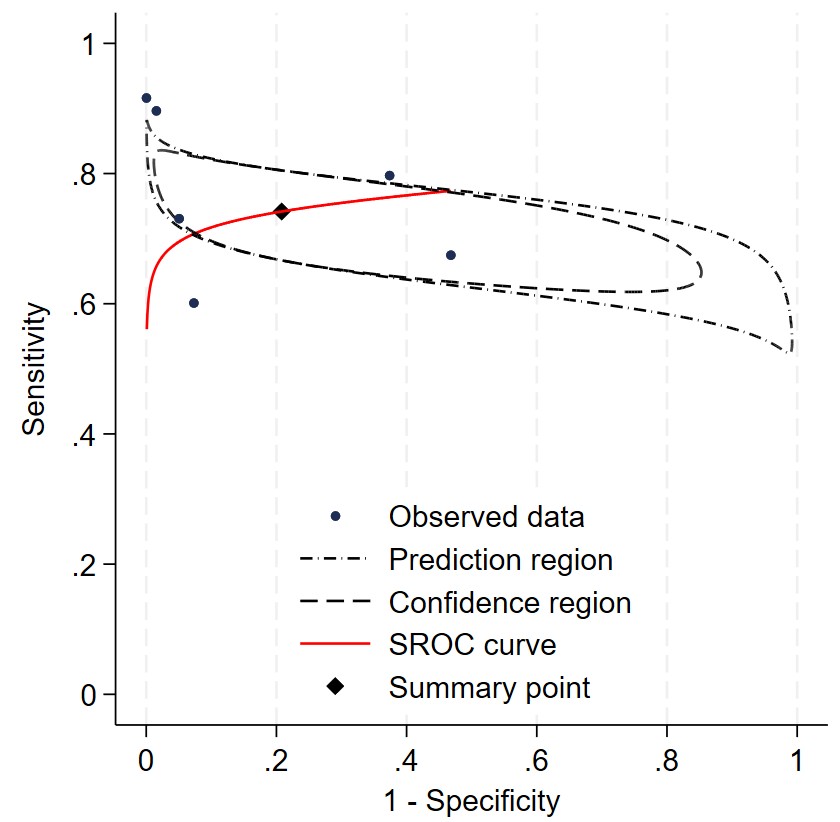 |
| --- |
| **S3.b. Funnel plot**  **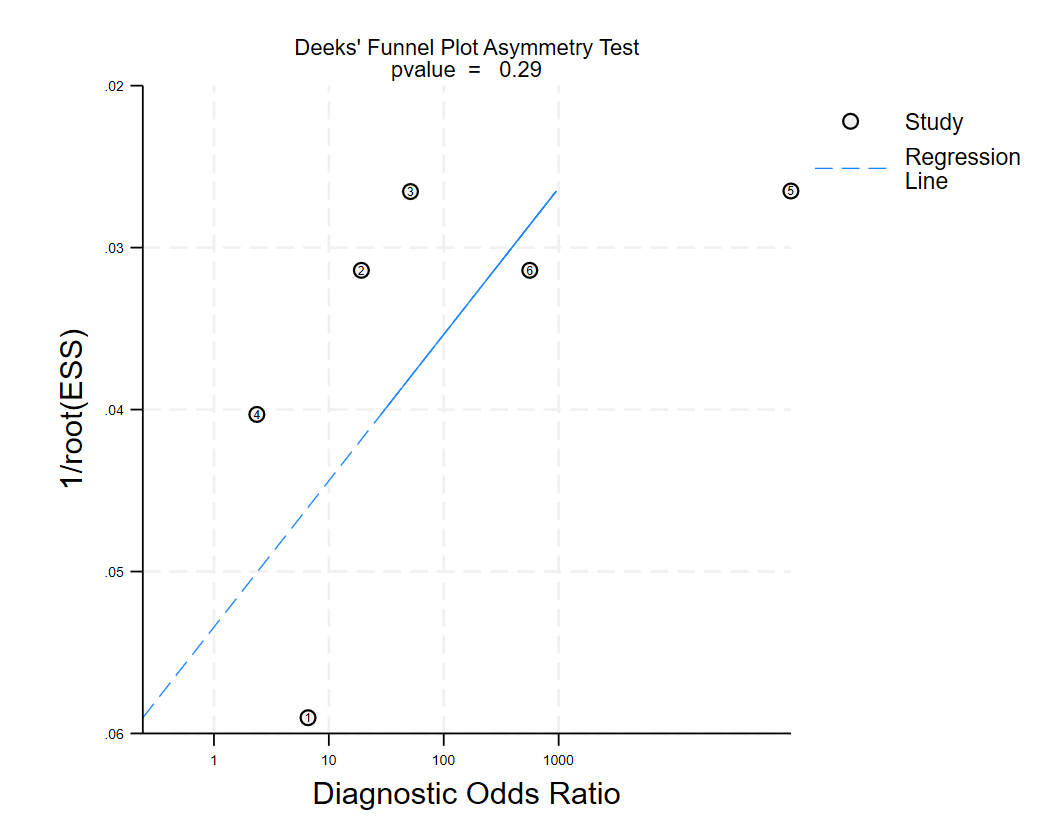** |
| **S3.c. Fagan’s plot**  **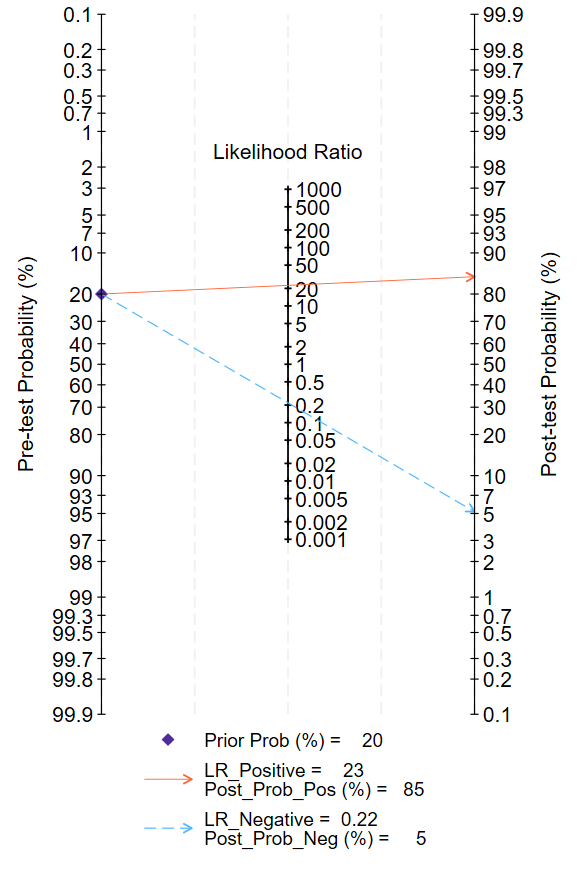** |
| **S3.d. Bivariate boxplot**  **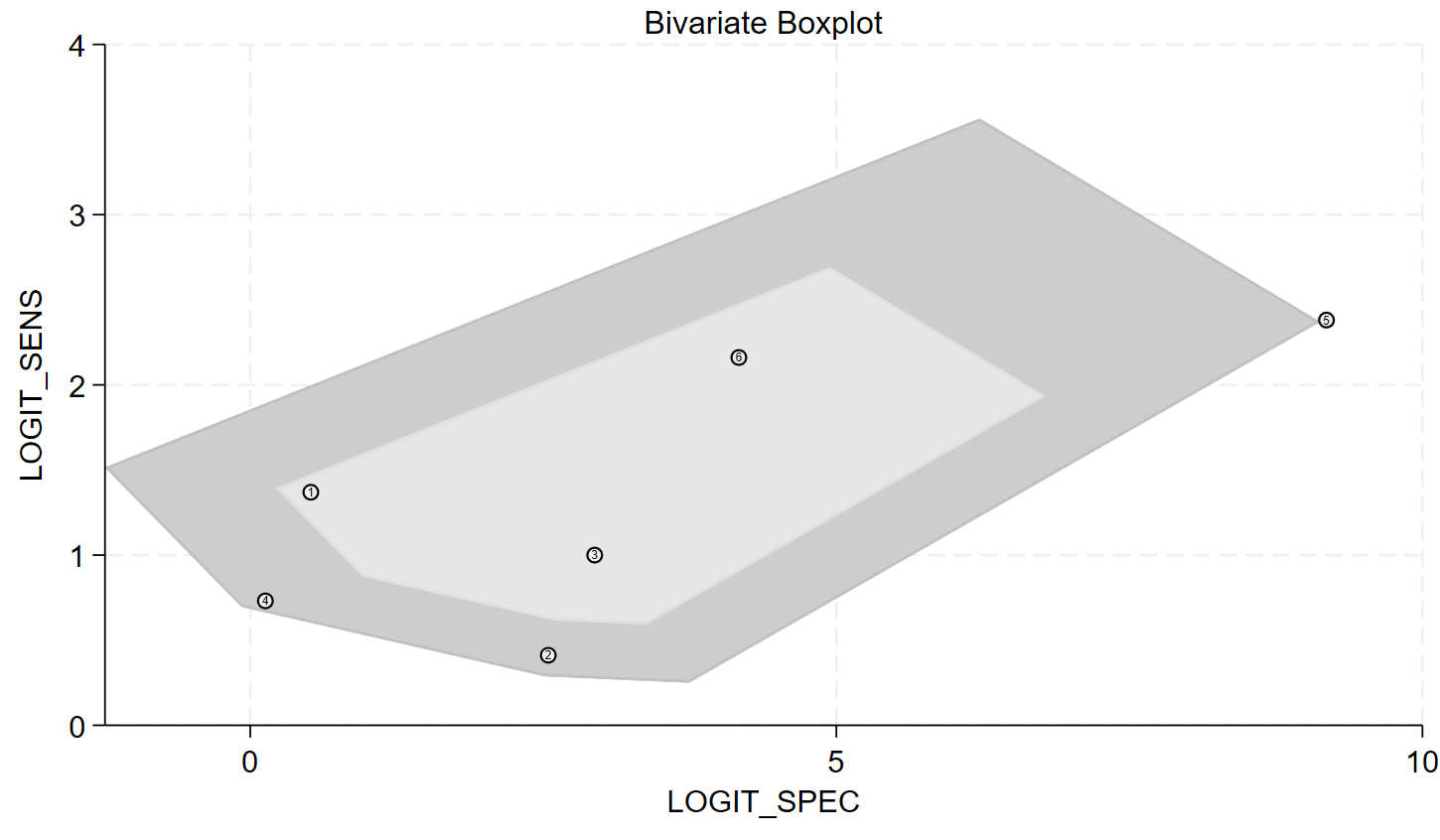** |
| **S3.e. Scatter matrix**  **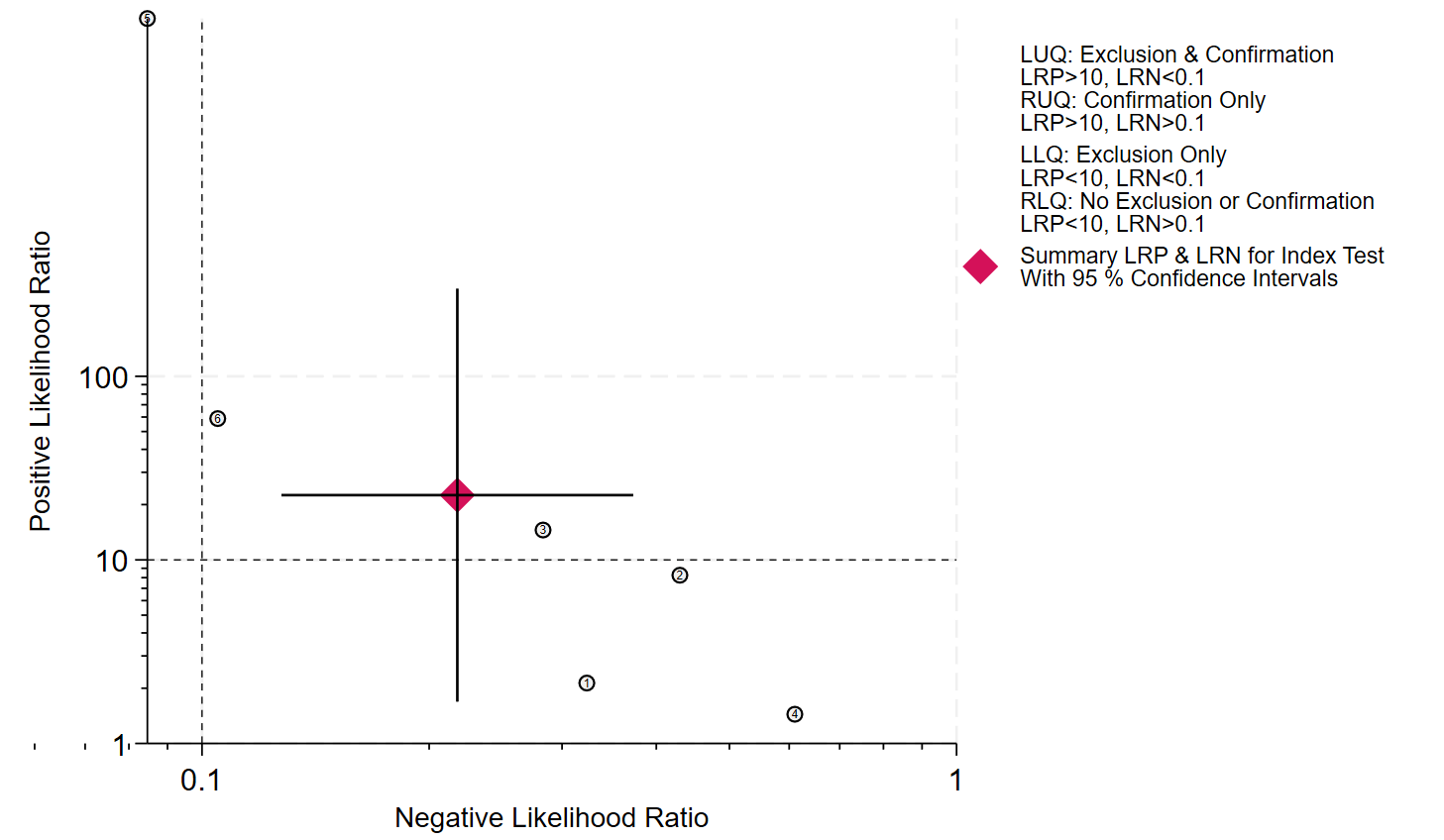** |

**Figure S4.** SCORE (Overall)

| **S4.a.** SROC  **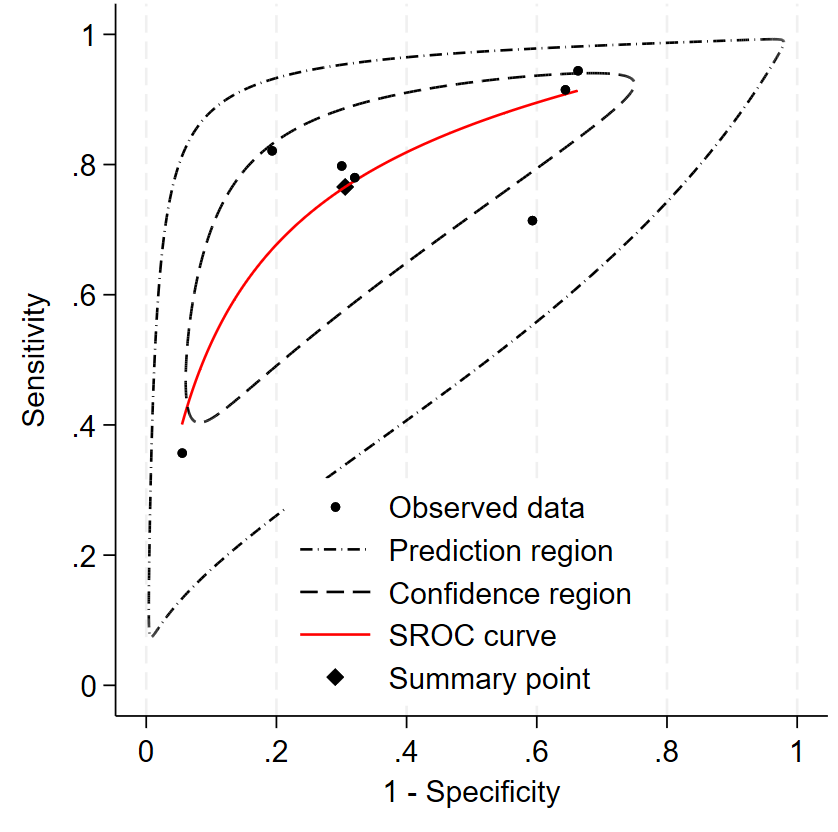** |
| --- |
| **S4.b. Funnel plot**  **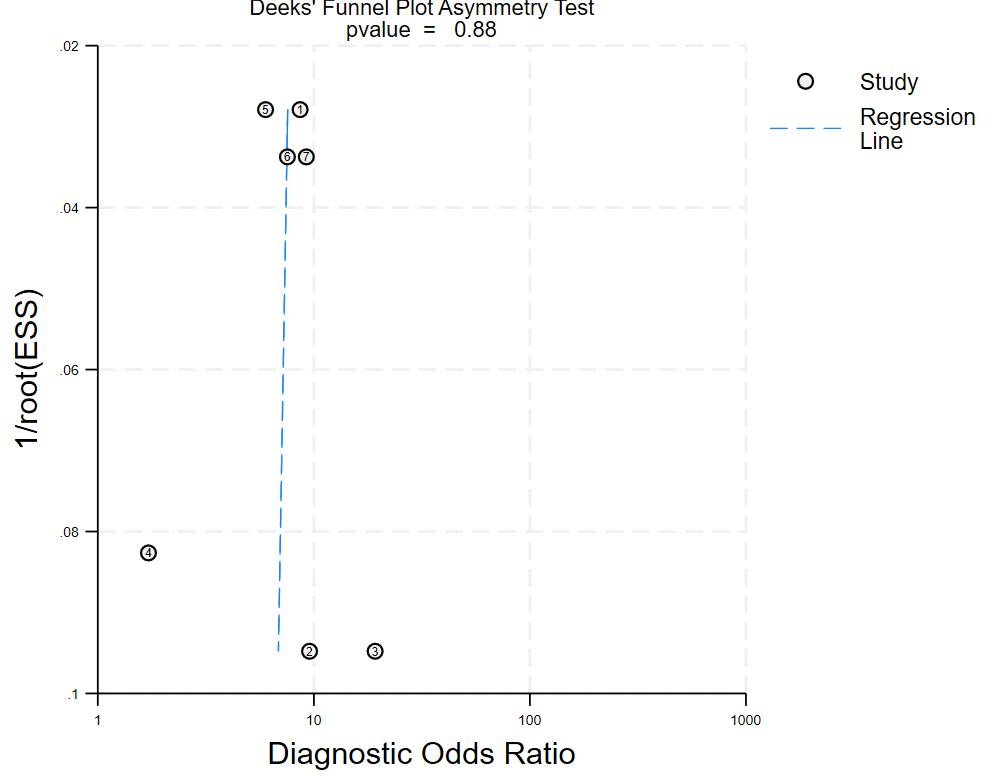** |
| **S4.c. Fagan’s plot**  **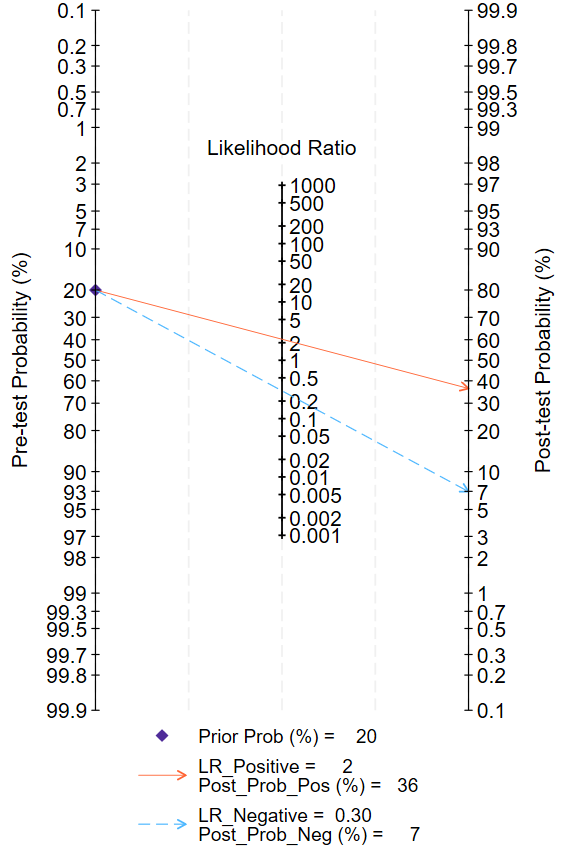** |
| **S4.d.** **Bivariate boxplot**  **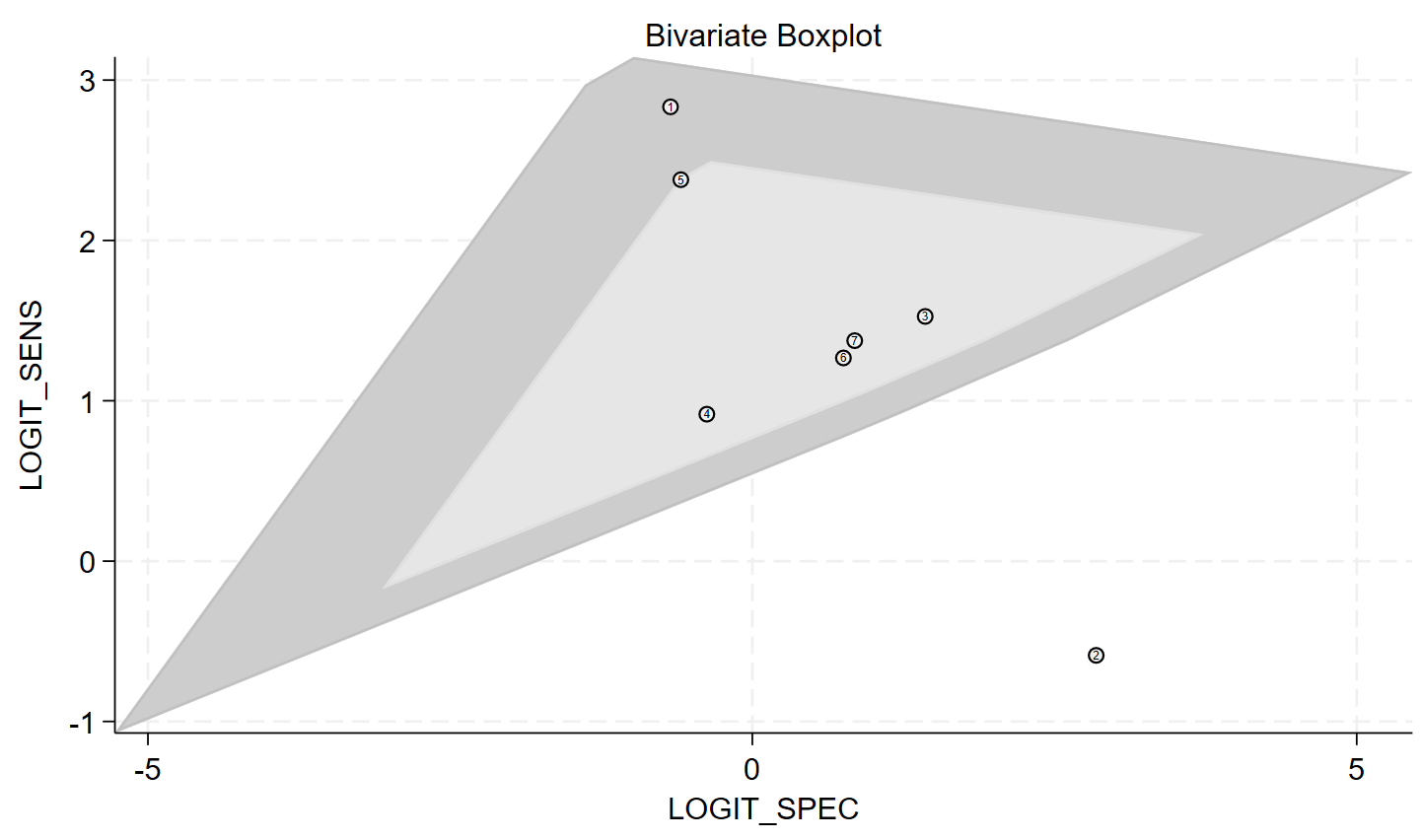** |
| **S4.e. Scatter matrix**  **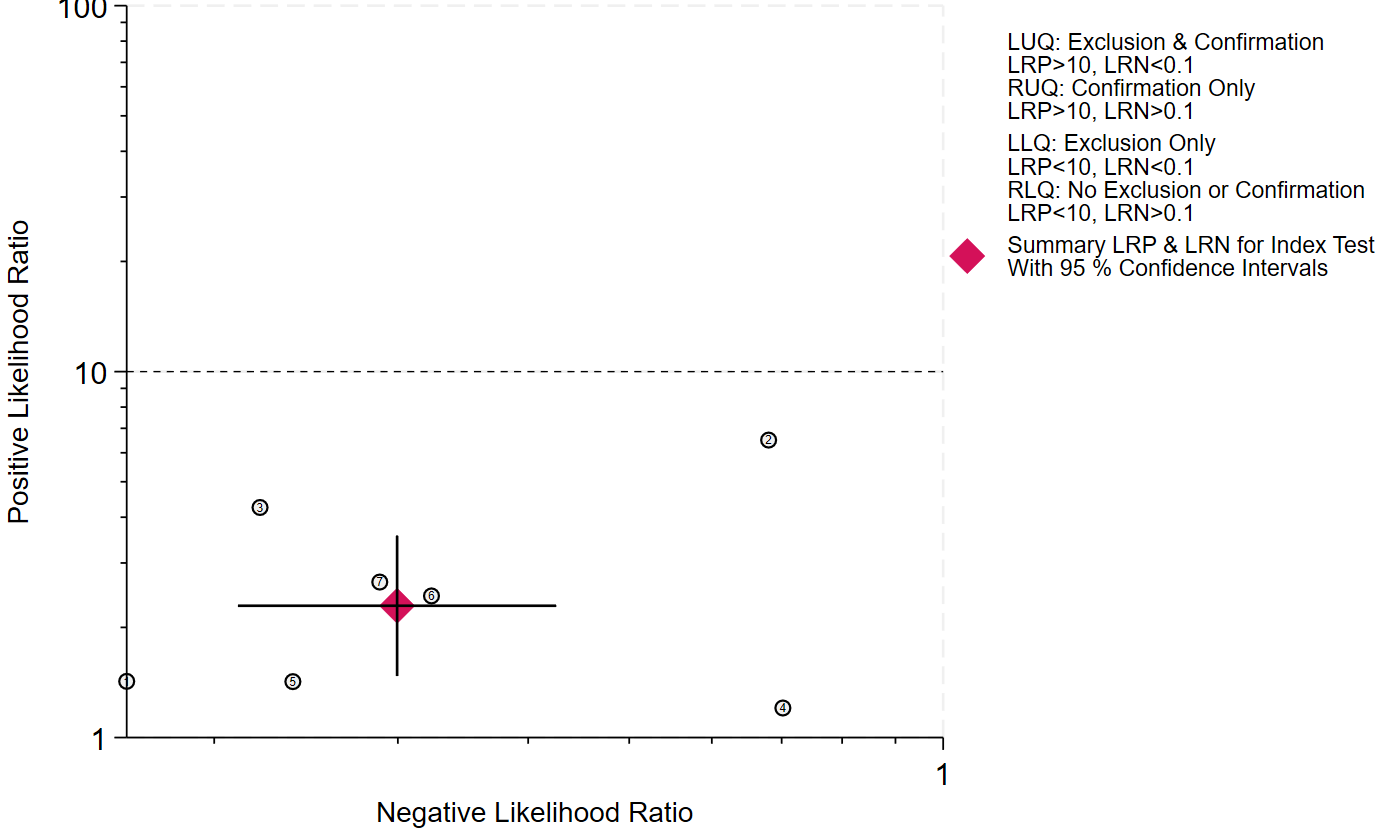** |

**(a) SROC curve:** The receiver operating characteristic (ROC) curve of prediction model of CVD, **(b) The Deek’s funnel-plot asymmetry test prediction model of CVD:** The horizontal axis represents the diagnostic odds ratio (DOR) as an indicator of the diagnostic accuracy, and the vertical axis represents the inverse of the square root of the effective sample size (1/root (ESS)). This figure shows symmetrical effect size measures (DOR) over different sample sizes, indicating no presence of publication bias, **(c) Fagan’s plot:** Positive LR: If the likelihood ratio is greater than 1, it indicates that a positive test result increases the probability of the disease. Negative LR: If the likelihood ratio is less than 1, it indicates that a negative test result decreases the probability of the disease. LR of 1: A likelihood ratio of 1 means the test result does not change the probability of the disease.

**(d) Bivariate boxplot:** Outliers: Studies falling outside the outer envelope are considered outliers, suggesting unusual combinations of sensitivity and specificity compared to the majority of studies. Heterogeneity: The plot helps visualize the heterogeneity in diagnostic performance across studies, revealing whether studies tend to cluster together or show a wide range of sensitivity and specificity values. Factors influencing accuracy: By examining the location of studies on the plot, researchers can investigate potential factors contributing to the observed variability, such as differences in study populations, thresholds used, or study designs.

**(e) Scatter matrix:** Upper Left Quadrant (+LR high, -LR low): Indicates a test that is good at both ruling in and ruling out the disease. This is the ideal quadrant. Upper Right Quadrant (+LR high, -LR high): Suggests the test is good at ruling in the disease but not as effective at ruling it out.

Lower Left Quadrant (+LR low, -LR low): Suggests the test is good at ruling out the disease but not as effective at ruling it in.

Lower Right Quadrant (+LR low, -LR high): Indicates a test that is neither good at ruling in nor ruling out the disease.

This scatter plot from a meta-analysis evaluates the diagnostic accuracy of an index test. The x-axis represents the negative likelihood ratio (LRN), and the y-axis represents the positive likelihood ratio (LRP). The plot is divided into four quadrants with thresholds at LRP = 10 and LRN = 0.1:

- **LUQ (Top-Left)**: LRP > 10, LRN < 0.1 – Indicates tests that are highly effective for both confirming (high LRP) and excluding (low LRN) a condition.
- **RUQ (Top-Right)**: LRP > 10, LRN > 0.1 – Suggests tests good for confirmation but poor for exclusion.
- **LLQ (Bottom-Left)**: LRP < 10, LRN < 0.1 – Indicates tests effective for exclusion but not confirmation.
- **RLQ (Bottom-Right)**: LRP < 10, LRN > 0.1 – Suggests limited diagnostic utility for both confirmation and exclusion.

The central diamond marker (LRP ≈ 10, LRN ≈ 0.1) is the summary point for the index test, showing it is on the boundary of significant diagnostic performance

**Figure S5.** The source of heterogeneity for FRS model. Bivariate boxplot


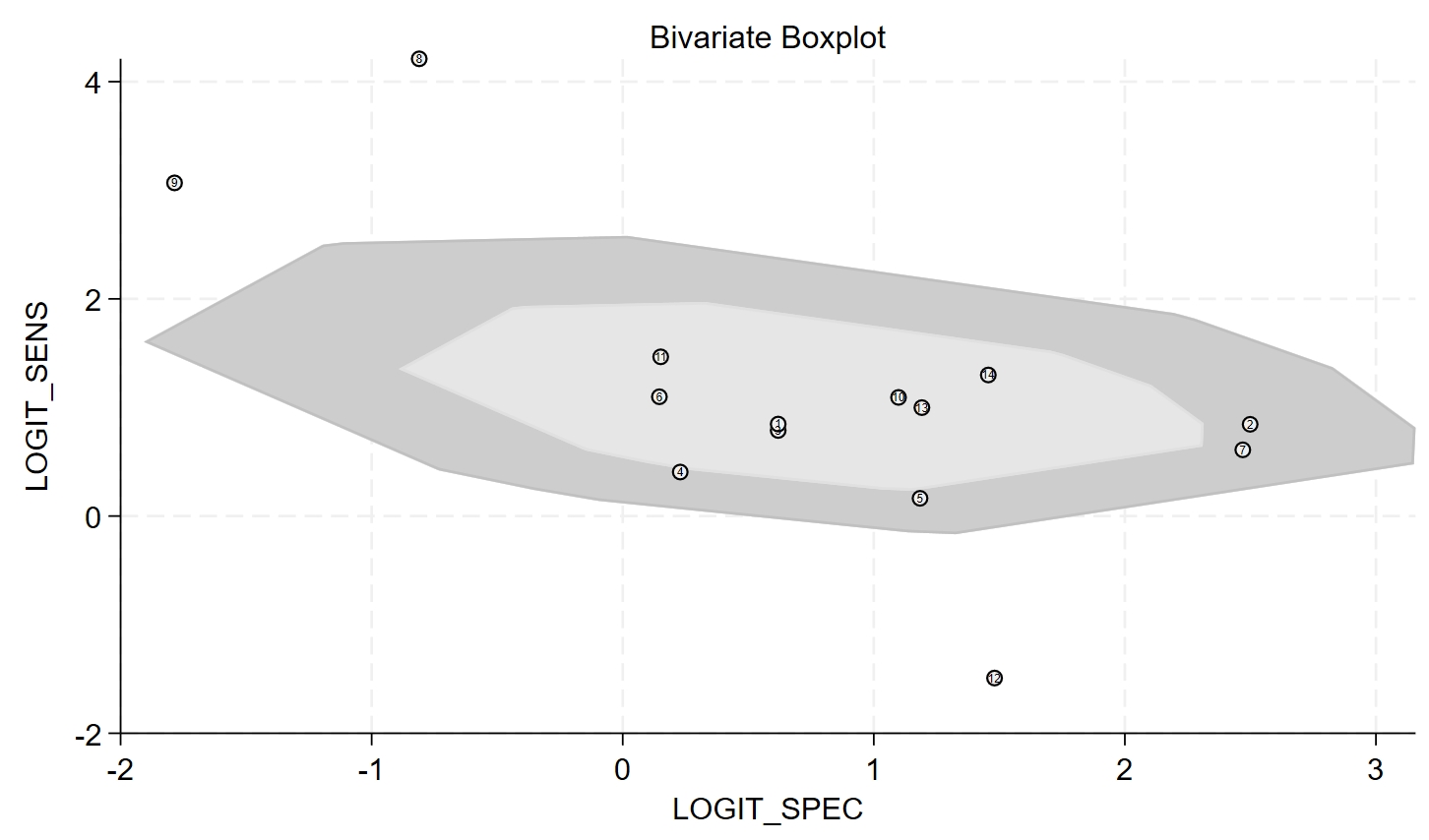

Supplement: Supplementary file 1 — Supporting File [file HSR2-9-e72469-s001.docx]
